# Characterisation and comparison of bacterial communities on reverse osmosis membranes of a full-scale desalination plant by bacterial 16S rRNA gene metabarcoding

**DOI:** 10.1038/s41522-017-0021-6

**Published:** 2017-06-19

**Authors:** Veena Nagaraj, Lucy Skillman, Goen Ho, Dan Li, Alexander Gofton

**Affiliations:** 1 School of Engineering and Information Technology, Murdoch University, Murdoch, Western Australia 6150 Australia; 2 School of Veterinary and Life Sciences, Murdoch University, Murdoch, Western Australia 6150 Australia

**Keywords:** Biofilms, Next-generation sequencing, Microbiome, Water microbiology, Applied microbiology

## Abstract

Microbiomes of full-scale seawater reverse osmosis membranes are complex and subject to variation within and between membrane units. The pre-existing bacterial communities of unused membranes before operation have been largely ignored in biofouling studies. This study is novel as unused membranes were used as a critical benchmark for comparison. Fouled seawater reverse osmosis membrane biofilm communities from an array of autopsied membrane samples, following a 7-year operational life-span in a full-scale desalination plant in Western Australia, were characterised by 16S rRNA gene metabarcoding using the bacterial primers 515F and 806R. Communities were then compared based on fouling severity and sampling location. Microbiomes of proteobacterial predominance were detected on control unused membranes. However, fouled membrane communities differed significantly from those on unused membranes, reflecting that operational conditions select specific bacteria on the membrane surface. On fouled membranes, *Proteobacteria* were also predominant but families differed from those on unused membranes, followed by *Bacteriodetes* and *Firmicutes*. *Betaproteobacteria* correlated with stable, mature and thick biofilms such as those in severely fouled membranes or samples from the feed end of the membrane unit, while *Alpha* and *Gammaproteobacteria* were predominantly found in biofilms on fouled but visually clean, and moderately fouled samples or those from reject ends of membrane units. *Gammaproteobacteria* predominated the thin, compact biofilms at the mid-feed end of membrane units. The study also supported the importance of *Caulobacterales* and glycosphingolipid-producing bacteria, namely *Sphingomonadales, Rhizobiales* and *Sphingobacteriia*, in primary attachment and biofilm recalcitrance. Nitrate-and-nitrite-reducing bacteria such as *Rhizobiales*, *Burkholderiales* and some *Pseudomonadales* were also prevalent across all fouled membranes and appeared to be critical for ecological balance and biofilm maturation.

## Biofouling: Biofilms and microbes on desalination membranes

The diverse microbial populations on seawater desalination plant membranes have been characterised after full operational lifecycles. The membranes were used for seven years to purify water by reverse osmosis. Biofouling can seriously impair the efficiency of the membranes but the problem has not previously been well characterised, especially after a full life-span of membrane operation. Veena Nagaraj and colleagues at Murdoch University in Australia investigated biofilms and used genetic analysis to identify the bacteria growing on 14 used membranes, and compared the results with pre-existing contamination on unused membranes. The research revealed that operational conditions favour the growth of specific bacterial populations, predominantly *Proteobacteria*, but also *Bacteriodetes* and *Firmicutes*. The results should assist research to devise new methods to prevent and alleviate the biofouling of desalination plant membranes and maximise the efficiency of their operation.

## Introduction

Bacteria exist as communities in biofilms of engineered systems such as desalination plants, incurring huge economic losses. Conditions on seawater reverse osmosis (SWRO) membrane surfaces select not just the most resistant organisms with superior attachment and biofilm forming abilities, but also an entire community that undergoes dynamic shifts in populations based on oxygen, nutrients and salt concentrations at different locations along the entire unit.^1^ The biofilm community may be described as diverse groups of colonists, with each group possessing a different set of skills and interacting rather than competing with each other to bring about an end result of resistant, mature biofilms that are almost impossible to dislodge. It is still not well understood where these bacteria originate from and how they access, attach and grow on membranes despite rigorous pre-filtration steps and regular cleaning procedures. To design efficient control measures, it is important to better understand how physiology and behaviour of predominant bacterial groups contribute to formation of recalcitrant biofilms under harsh conditions within desalination plants.

To date no 16S rRNA gene metabarcoding studies have been published on characterisation of aged, mature biofilm communities from various locations within full-scale SWRO membrane units after completion of their life-span. However, a handful of published studies conducted on membranes of full-scale desalination plants involved younger biofilms aged 1 year or less.^2, 3^ The long life-span (>7 years) of SWRO membranes in full-scale systems, and difficulty of access to sealed membrane units and sampling from an operational plant are the likely reasons for only a few studies. Biofouling has been known to occur within the first few hours of starting the SWRO operation.^4, 5^ As most manufacturing processes are not sterile, it is not known if biofouling organisms inhabit membrane surfaces prior to operation. Bacterial populations on unused membranes are critical benchmarks for comparison with populations on fouled SWRO membranes. Studies on bacterial communities colonising clean membrane surfaces prior to their use in the full-scale plant have not been published to date.

The objectives of our study were to characterise and compare bacterial communities on SWRO membranes procured from a full-scale desalination plant in Western Australia. Membranes were obtained from the plant at the end of their functional life of 6 to 7 years, when SWRO units were being replaced by new membrane units. In order to better understand variations in microbial community ecology, fouled membrane samples were grouped into different categories, namely, a) severity of fouling; and b) membrane location along the course of the unit. Bacterial communities on unused membranes were also compared to those of differing fouling severity. Communities were also compared to understand how operational conditions affect ecological balance along the course of membrane units from the feed end to the reject end. The above comparison was necessary to establish the choice of best model sampling sites for future studies to ensure that the sample biofilm community was a good representation of the entire microbial community in the plant.

This study presents interesting findings about the membrane biofilms, their major bacterial groups and the changes in the composition of the groups which reflect the ecological shifts in the biofouling community of full-scale SWRO membranes. Variations in bacterial diversity at different locations within each SWRO unit also reveals bacterial adaptation to changes in permeate flux, feed-water flow and salt rejection rates.

## Results

**Fig. 1:**
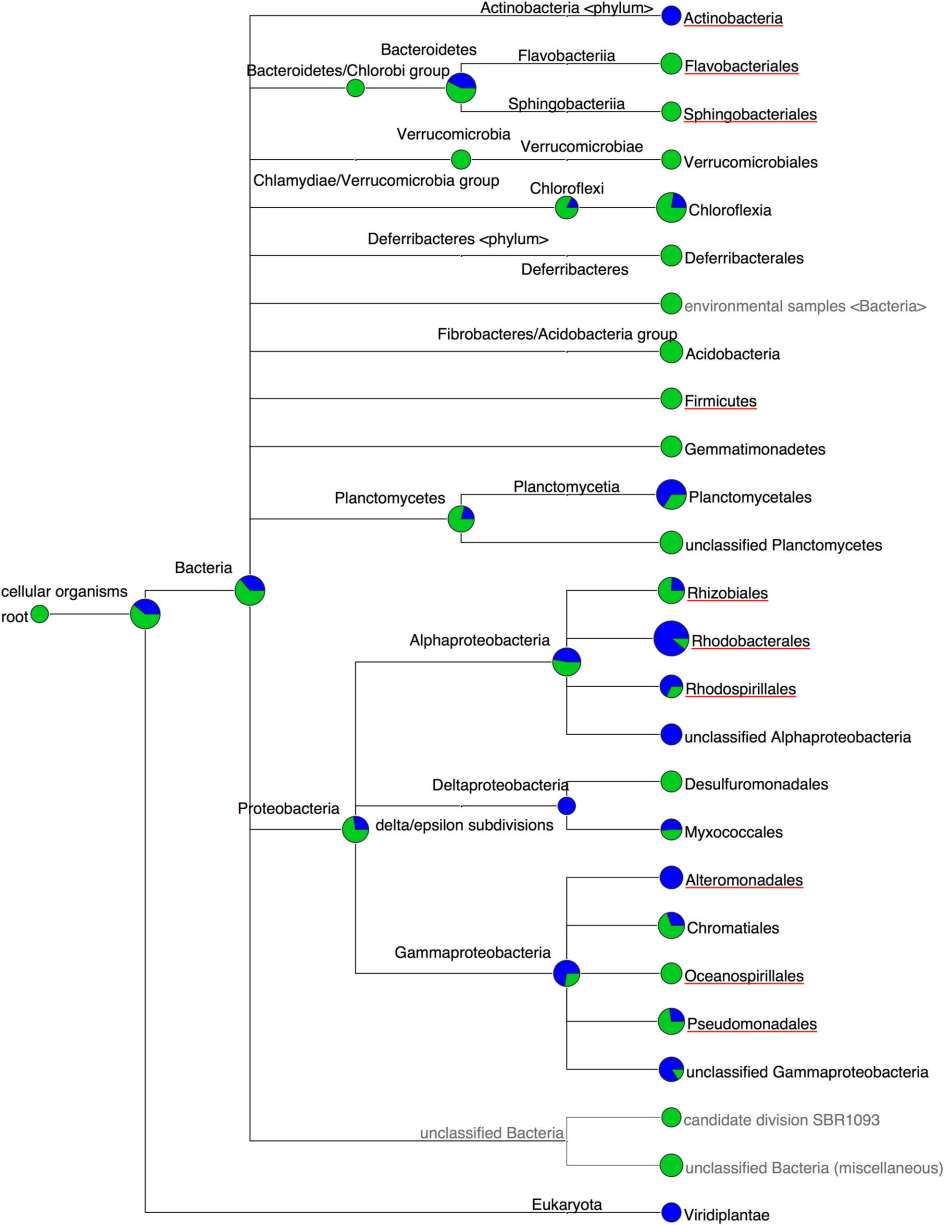
MEGAN cladogram representing the bacterial community composition of feed-water (*green dot* *s*) and polished seawater (*blue dots*) from the Perth Seawater Desalination Plant. The underlined taxa represent bacterial orders that were present in the RO-membrane biofilm community

The results of the preliminary study that included analysis of bacterial communities in source water and polished seawater are presented in Fig. 1. The bacterial community of feed-water comprised of a high proportion of *Flavobacteriaceae, Firmicutes* and *Pseudomonadaceae*, while the polished seawater was high in the abundance of *Actinobacteria*, *Alteromonadaceae* and *Rhodobacteriaceae* (Fig. 1).

### Biomass volume and biofilm depth

**Fig. 2:**
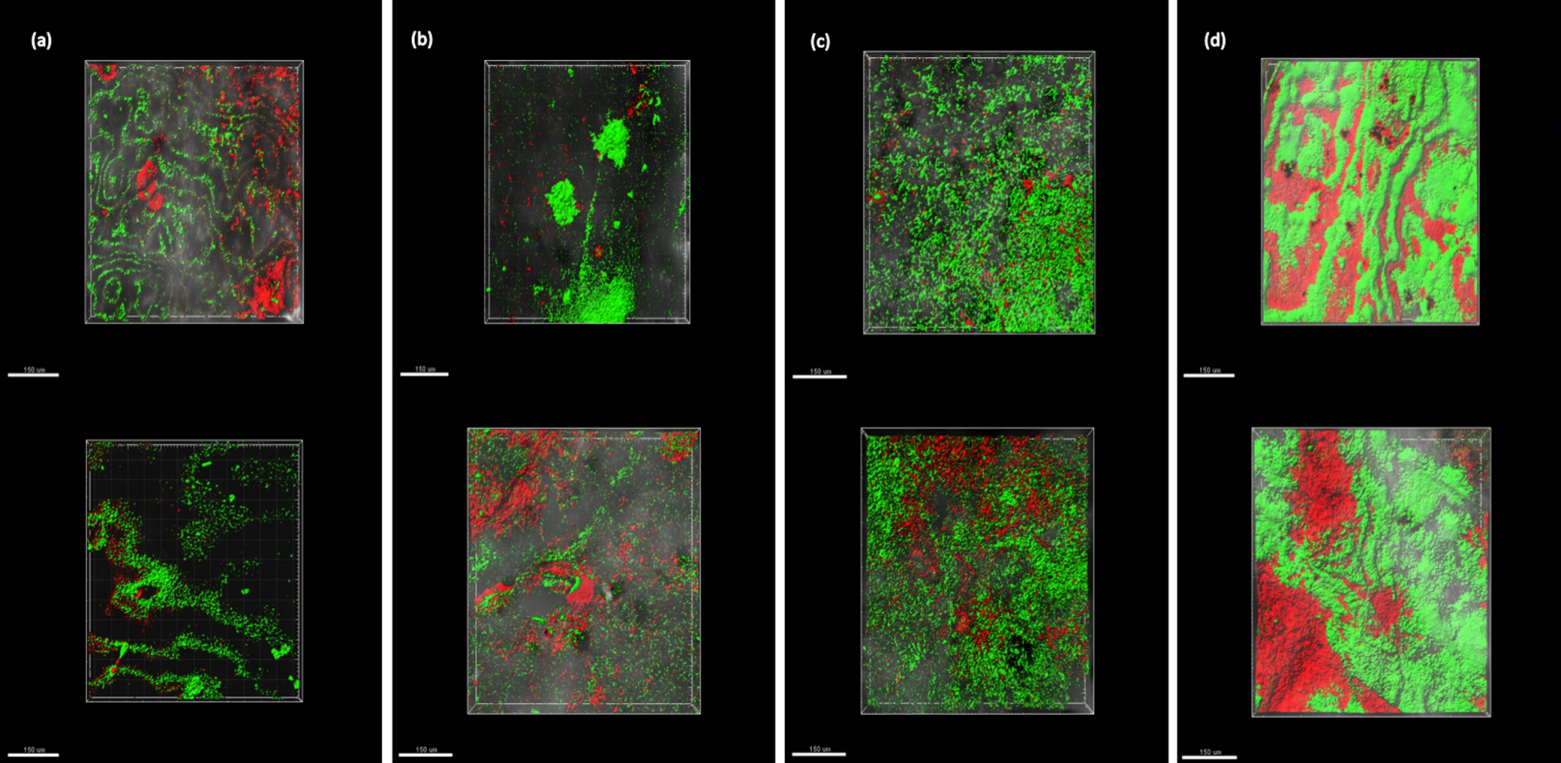
Reconstructed CSLM images of live/dead-cell-stained biofilms on the unused control RO membranes - Biomass volume 3.15  ±  0.06  µm^3^ and Biofilm depth 30  ±  2 µm (**a**); clean RO membranes - Biomass volume 2.72  ±  0.28  µm^3^ and Biofilm depth 62  ±  22  µm (**b**); moderately fouled RO membranes - Biomass volume 4.04  ±  0.49  µm^3^ and Biofilm depth 51  ±  21  µm (**c**); and severely fouled RO membranes - Biomass volume 6.16  ±  3.35  µm^3^ and Biofilm depth 64  ±  16  µm (**d**)

Biofilms on the unused RO membranes were the thinnest among all membrane biofilms, as expected; however, their biomass volume was higher than that of the fouled but visually clean membranes. The average biomass volume (µm^3^) varied between membranes, depending on the severity of fouling (Fig. 2). Among fouled membranes, the moderately fouled membranes had a low biofilm depth in relation to their relatively higher biomass volume, suggesting that biofilms were more dense/compact compared to the clean and severely fouled membranes. The average biomass volume of feed, middle and reject end of membranes was 4.44 ± 2.40 µm^3^ each, and that of the mid-feed region was 3.86 ± 1.91 µm^3^. The average biofilm depth of the feed, mid-feed, middle and reject end of membranes were recorded as 63.7 ± 15 µm, 36.0 ± 8 µm, 47 ± 9 µm and 58.6 ± 18 µm, respectively. Biofilms were thinnest and most compact at the mid-feed end, increasing in thickness towards the middle region of the membranes.

### DNA extraction and NGS metrics

**Fig. 3:**
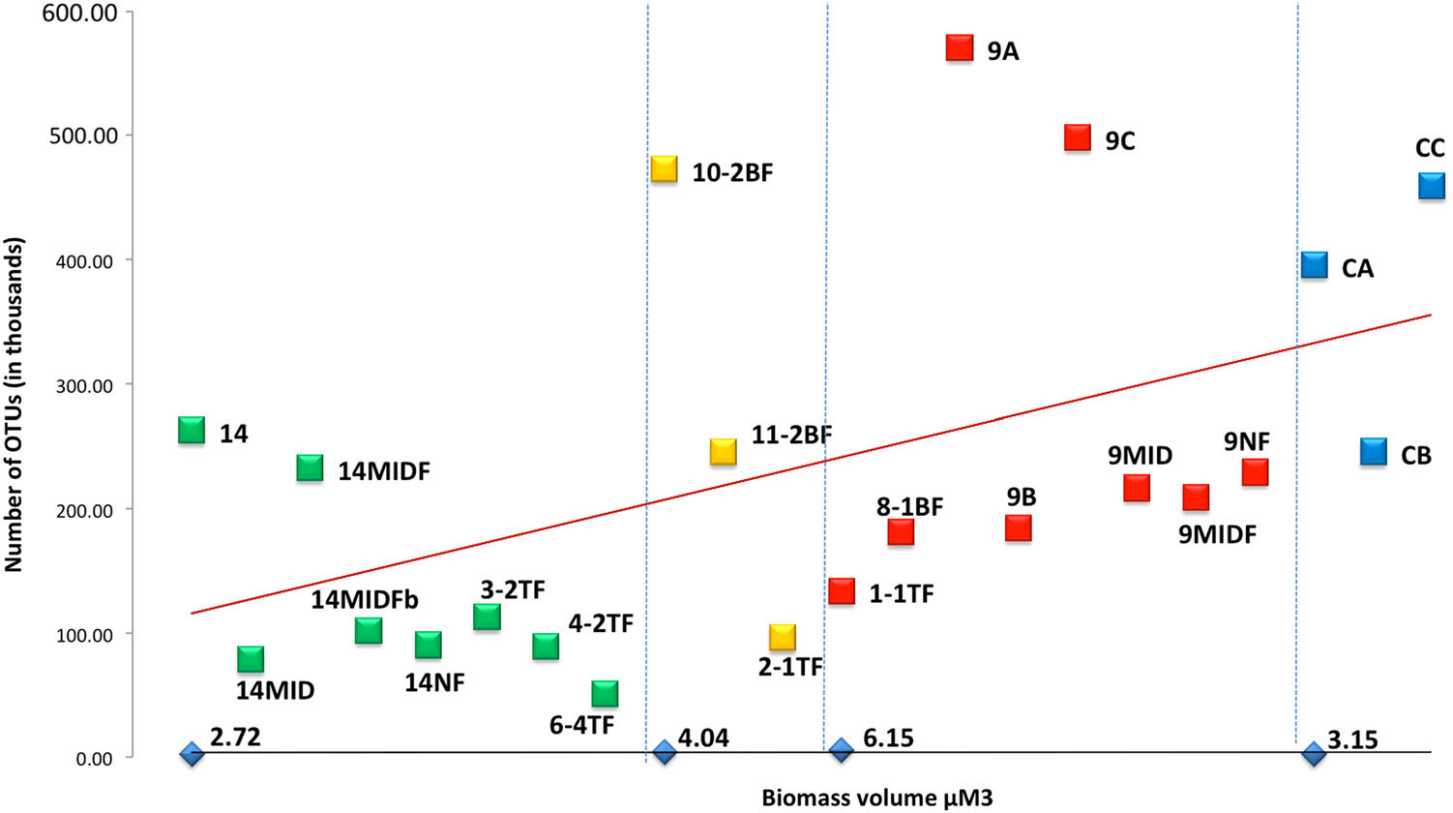
Linear regression graph of the total number of OTUs sequenced from the clean (*green*), moderately fouled (*yellow*), severely fouled (*red*), and unused control RO membranes (*blue*), against the average biofilm-biomass of the corresponding groups of membranes. The *vertical blue dotted lines* separate the different classifications of membranes

The most important prerequisite for community diversity profiling using DNA-based sequencing methods is sufficient high quality DNA. The DNA extraction method using the PowerSoil^®^ DNA Isolation Kit (MO BIO, Qiagen) resulted in low yields of DNA ranging from 0 to 8 ng/μL. The modified phenol-chloroform extraction method resulted in effective DNA extraction from all samples with quantities ranging from 32 to 250 ng/μL. The UV absorption peaks at *A*_260/280_ ranged from 1.58 to 1.76 and indicated that the method generated relatively clean DNA. The yield of DNA for all membrane samples are presented in Table 1 and the number of OTUs sequenced, in relation to their biofilm-biomass are presented in Fig. 3.

**Table 1:** Classification of membrane samples, yield of DNA and total number of OTUs per sample

Sample ID	Condition	Location	Extraction replication	Yield of DNA (ng/µL)	Total number of OTUs
14	Clean	Feed end		104.3	263012
14MID	Clean	Middle		102.4	78457
14MIDF	Clean	Mid-feed end		46.1	232817
14MIDFb	Clean	Mid-feed end		220.4	102199
14NF	Clean	Reject end		170.1	90239
3-2TF	Clean			136	112542
4-2TF	Clean			94.3	89422
6-4TF	Clean			74.4	51237
10-2BF	Average (moderately fouled)			249.6	472899
11-2BF	Average (moderately fouled)			74.5	245562
2-1TF	Average (moderately fouled)			174.2	95910
1-1TF	Dirty (severely fouled)			148.7	133766
8-1BF	Dirty (severely fouled)			49.7	180978
9A	Dirty (severely fouled)	Feed end	Fouled membrane	52.2	570673
9B	Dirty (severely fouled)		Fouled membrane	52.9	184556
9C	Dirty (severely fouled)		Fouled membrane	154.7	498320
9MID	Dirty (severely fouled)	Middle		179.5	216617
9MIDF	Dirty (severely fouled)	Mid-feed end		136.5	208750
9NF	Dirty (severely fouled)	Reject end		95.3	229328
CA	Unused		Control	30.9	395766
CB	Unused		Control	38.3	245542
CC	Unused		Control	62.1	459179

The total number of OTUs sequenced from the membrane samples of varying severity of fouling was directly proportional to their biomass volume, with a range of 51–263K, 96–473K and 134–571K OTUs from clean, moderately fouled and severely fouled membranes, respectively (Fig. 3). Amplification of DNA from the middle or reject end of clean membranes (*n* = 6/22) resulted in the lowest number of sequences in the range 51–113K. Clean membrane samples from the feed end (14) and mid-feed end (14MIDF) (*n * = 2/22) yielded a higher number of OTUs in the range 233–263K, which may be due to a greater biofilm thickness compared to the middle and reject ends (Fig. 3). Moderately fouled membrane samples (*n* = 3/22) yielded a wide range of OTUs (96–473K). They were all sampled from the feed end. As expected, the feed end samples of severely fouled membranes (9A, 9C) yielded the highest number of OTUs (498–571K) compared to the number of OTUs recovered from feed end samples of clean and moderately fouled membranes. The control unused membranes yielded a relatively high number of OTUs ranging from 246–459K in relation to their biomass volume.

To check if each sample was sequenced with sufficient depth to reliably determine the entire microbial community of each sample, alphararefaction was calculated based on alpha diversity. All samples including the control unused membranes showed a plateau indicating that the sequencing depth was sufficient to represent the diversity of all genera present in the samples (Supplementary Fig. 1).

**Fig. 4:**
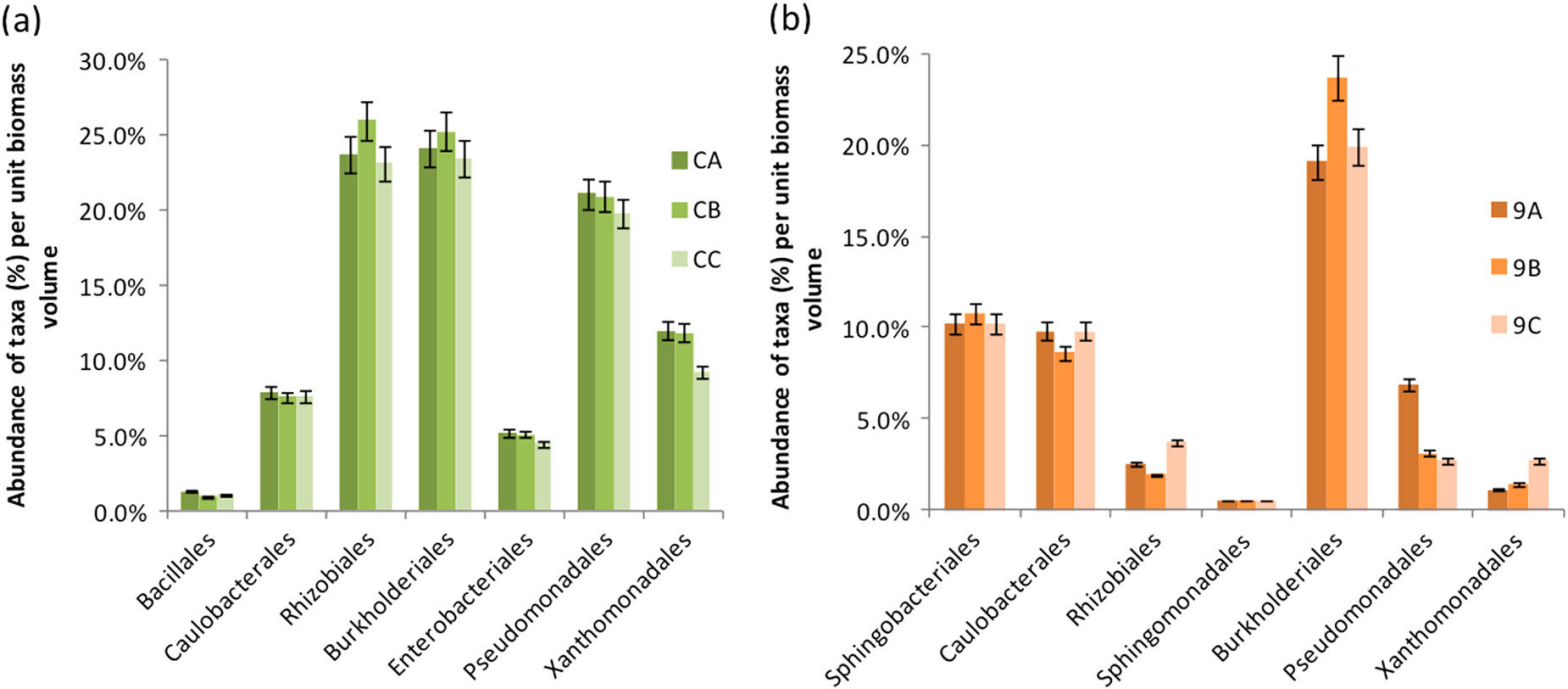
Abundance of major taxonomic orders (%) per unit biomass volume of RO-membrane-biofilm in replicate samples of unused control membranes (**a**) and fouled membranes (**b**) *Error bars* represent percent SE

Upon comparison of the abundance of major taxa present on individual membrane samples of the unused (CA, CB, CC) and the fouled membrane (9A, 9B, 9C) control groups, it was evident that there was minimal variation between replicate samples within the groups (Fig. 4). This suggested the similarity in bacterial community structure between replicate samples that belonged to the same membrane. *Rhizobiales*, *Burkholderiales* and *Pseudomonadales* were among the most abundant in the unused control membrane group while *Burkholderiales* were the most abundant in the fouled membrane sample replicates.

**Fig. 5:**
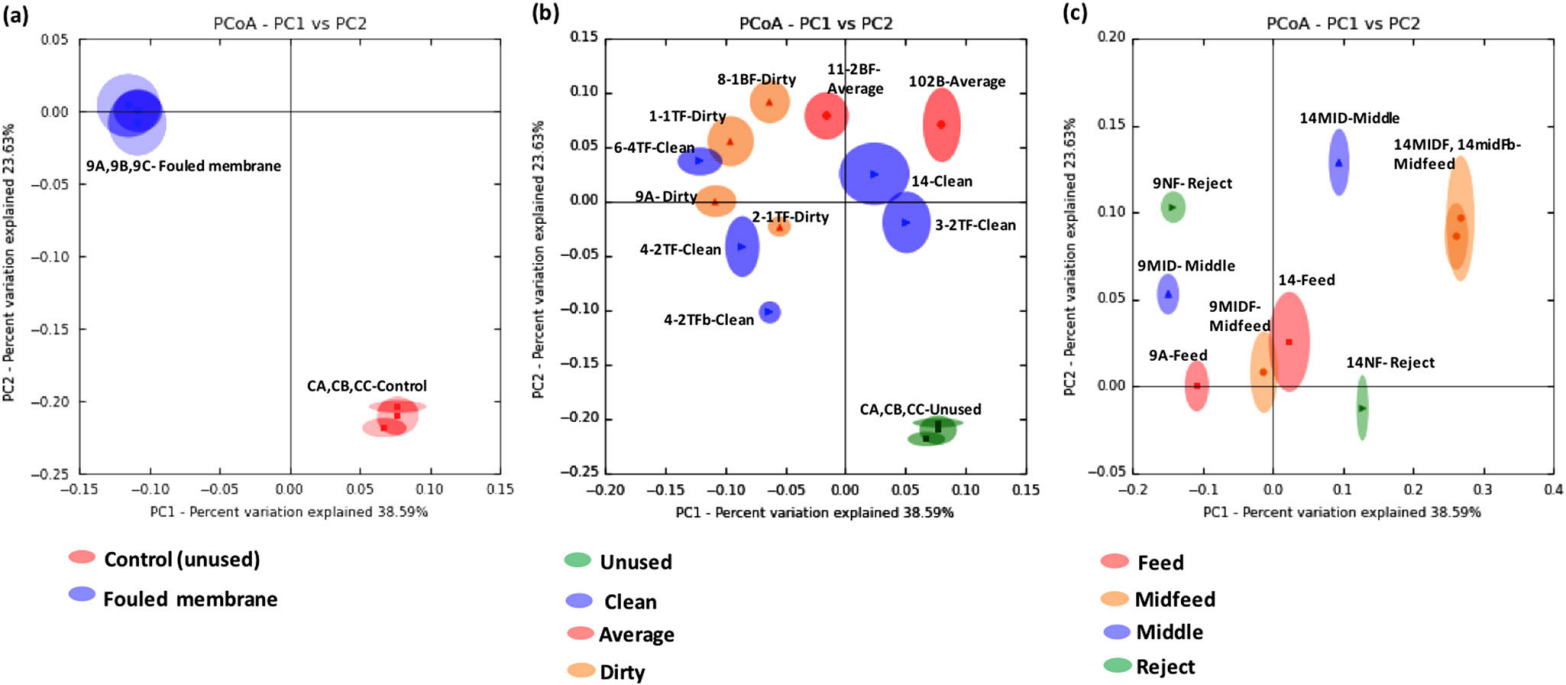
Jackknifed PCoA analysis using weighted Unifrac distances of bacterial communities in the extraction replicates of control and fouled RO membranes (**a**); unused, clean, moderately fouled (average) and severely fouled (dirty) RO membranes (**b**); feed, mid-feed, middle and reject end of RO membranes (**c**)

In the jackknifed PCoA analysis, bacterial communities from replicate samples of unused membranes (CA, CB, CC) clustered together. Similarly, replicates of severely fouled membranes (9A, 9B, 9C) clustered close to each other (Fig. 5a). This indicated that the replicate extracts were most closely related to each other, suggesting sufficient quantities of DNA were recovered from each sample to effectively represent the membrane biofilm community. It also suggested that the replicates from different sections of the same membrane were the same, thus validating the sampling approach.

### Comparison of bacterial communities between unused membranes and fouled membranes with differing severity of fouling

**Fig. 6:**
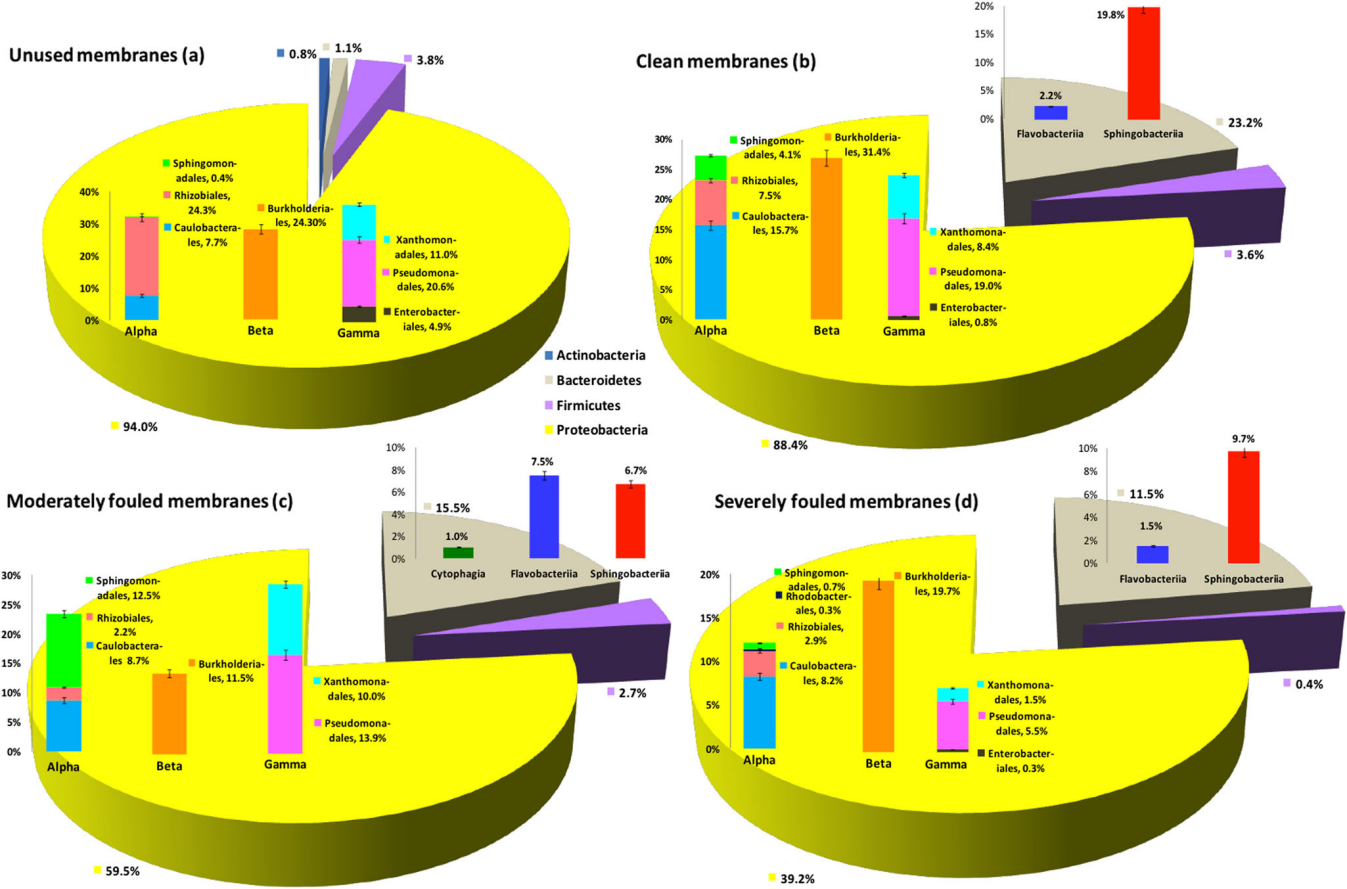
Abundance of major bacterial taxa (%) per unit biomass volume of RO-membrane-biofilm, present in the unused (**a**), clean (**b**), moderately fouled (**c**) and severely fouled (**d**) RO membranes. *Error bars* represent percent SE

The abundance of major bacterial taxa, calculated as a percentage per unit of biofilm-biomass volume are compared in Fig. 6, and the actual abundance of taxonomic groups, are presented as Krona plots^6^ in Supplementary Fig. 2. There were key similarities and differences between communities of unused and fouled membranes (Fig. 6). At the phylum level, both unused and all categories of fouled membranes were dominated by *Proteobacteria*. However, there was an increased abundance of *Bacteriodetes* in fouled membranes (*n*  = 11/14) compared to unused samples (*n*  = 3/14). Among the *Proteobacteria*, *Alpha* and *Betaproteobacteria* were the most abundant in the clean membranes (*n * = 5/14) (Fig. 6b). The *Betaproteobacteria*, were almost entirely *Burkholderiales*, which also were dominant members of severely fouled membranes (*n* = 4/14) (Fig. 6d), while the *Gammaproteobacteria* dominated the bacterial community in unused (*n * = 3/14) and moderately fouled membranes (*n* = 2/14) (Fig. 6a, c). Within the *Bacteriodetes* phylum, *Sphingobacteriia* were of highest abundance in both clean membranes and severely fouled membranes (Fig. 6b, d). However, on the moderately fouled membranes, the population of *Flavobacteriia* increased to equal the abundance of *Sphingobacteriia* within the phylum (Fig. 6c).

With respect to the actual abundance, *Ochrobactrum* (21%) was the most abundant genus followed by *Pseudomonas* (18%), *Delftia* (13%) and *Stenotrophomonas* (10%) in the unused membranes (Supplementary Fig. 2a). Among these, *Pseudomonas* was also common to all fouled membrane samples (Supplementary Fig. 2b, c, d). The genera *Caulobacter, Undibacterium, Sphingobacter, Sphingopyxis* and *Pedobacter* were abundant among the bacterial communities in the fouled membranes, but were detected at very low levels in the unused membranes. Members of *Enterobacteriaceae*, namely *Escherichia/Shigella* were present up to an abundance of 5% in the unused membranes.

### Comparison of bacterial communities according to membrane location

**Fig. 7:**
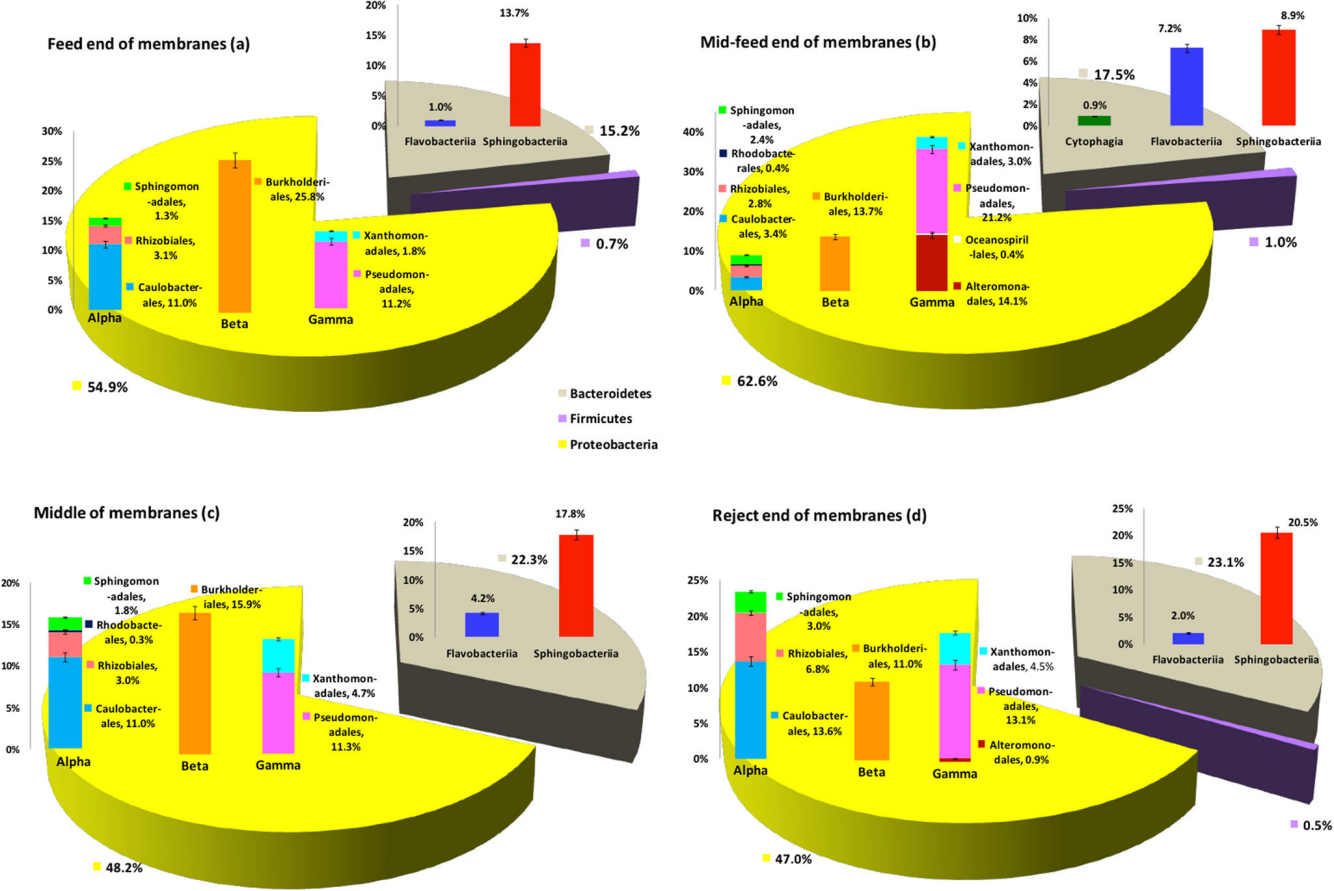
Abundance of major bacterial taxa (%) per unit biomass volume of RO-membrane-biofilm, present in the feed (**a**), mid-feed (**b**), middle (**c**) and reject (**d**) end of the membranes. *Error bars* represent percent SE

The key similarities and differences between bacterial communities of various membrane locations are presented in Fig. 7. Phylum distribution was similar across all locations, with proteobacterial sequences being the most abundant, followed by *Bacteriodetes*. Among the *Proteobacteria*, *Betaproteobacteria*, comprising entirely *Burkholderiales*, dominated at the feed end (*n* = 2/9) and middle of the membranes (*n* = 2/9) (Fig. 7a, c), while *Gammaproteobacteria* dominated the mid-feed (*n* = 3/9) end (Fig. 7b). *Alphaproteobacteria* were the most abundant at the reject end (*n* = 2/9) of the membranes (Fig. 7d). Among the *Bacteriodetes*, *Sphingobacteriia* dominated all locations while *Flavobacteriia* were also evident at a high abundance along with *Sphingobacteriia* in the mid-feed end of the membranes.

### Bacterial community alpha diversity estimates of bacterial communities on SWRO membranes

Alpha diversity is a measure of the mean species diversity of bacteria within each sample. The abundance and evenness or distribution of genera was evaluated using the Shannon–Wiener index H′ (Table 2; Supplementary Figures 3a, 4a, 5a), while the species richness of communities was calculated using the Chao1 index (Table 2; Supplementary Figures 3b, 4b, 5b).

**Table 2:** Diversity indices in different matrices among treatment groups of membranes

Treatment	Shannon–Wiener index H′	Chao1 index S
Extraction replication		
Unused control	3.68 ± 0.23	228.39 ± 21.44
Fouled membrane	3.83 ± 0.1	397.25 ± 20.55
Condition		
Moderately fouled	4.62 ± 0.2	406.9 ± 41.99
Clean	4.35 ± 0.31	321.37 ± 83.4
Severely fouled	4.06 ± 0.36	365.66 ± 20.71
Unused	3.67 ± 0.23	228.39 ± 21.44
Membrane location		
Feed	4.03 ± 0.15	427.97 ± 46.62
Mid-feed	4.17 ± 0.4	423.55 ± 46.1
Mid	4.04 ± 0	321.73 ± 30.79
Non-feed/reject	4.41 ± 0.29	407.54 ± 29.06

The Shannon diversity of extraction replicates within each of the unused (control) and the fouled membrane groups were similar, but there was a significant difference between species richness measured using the Chao1 index, with unused membranes having a much lower species richness than fouled membranes (Table 2).

As expected, bacterial alpha diversity was lowest in the unused membranes measured by the Shannon–Wiener index H^1^, while the clean, moderately fouled and severely fouled membranes had similar diversities (Table 2). Species richness was highest in the moderately fouled membranes and lowest in unused membranes. However, the clean and severely fouled membranes exhibited similar values of species richness. There was a significant difference between the species richness of unused and severely fouled membranes (*p* < 0.05) (Supplementary Table 1b).

The diversity (Shannon–Wiener H^1^-index) of bacterial communities was similar in membrane samples from the feed, mid-feed end, middle and non-feed/reject end of the membranes (Table 2) with no significant differences between them (Supplementary Table 1a). Species richness was highest at the feed end of membranes, followed by the mid-feed location, reject end and middle of membranes (Table 2). However, the differences in species richness were not significant (Supplementary Table 1b).

### Bacterial community beta diversity estimates of reverse osmosis membranes

Communities from unused membranes clustered close to each other but were well separated from communities of fouled membranes (Fig. 5b) with no overlaps between clusters of unused and fouled membranes in the jackknifed PCoA. The sampling number and depth was effective in representing the whole group of unused membranes, possibly due to the relatively smaller bacterial community inhabiting the unused membrane surface. Among fouled membranes, bacterial communities from the severely fouled (dirty) membranes were more closely clustered with each other while communities from the moderately fouled (average) and the clean membranes were more widely spread. The only exception in the spread of bacterial communities from the clean membranes was 14 Clean and 3-2TF Clean, which had overlapping regions in the PCoA. There were also overlaps between the bacterial communities of the dirty membrane 1-1TF and clean membrane 6-4TF (Fig. 5b), indicating that bacterial communities on these membranes with varying severity of fouling were closely related to each other and shared common genera. There was no significant difference between their diversities (*p * > 0.05).

In addition to sampling several membrane units (14), additional samples were taken from different locations of two membranes, one quite clean (14s) and one severely fouled (9s) (Table 1). Bacterial communities from clean samples of the mid-feed region (14MIDFb, 14MIDF) clustered together with overlaps. They were the most distant from all other communities indicating they were distinct from the other bacterial populations. Communities of the dirty membrane sample (9MIDF) and clean sample (14-Feed), both from the feed end also clustered closely exhibiting overlaps. Bacterial communities from all other membrane samples, assigned to the treatment group of sampling location, did not form clusters and were more widely spread (Fig. 5c). There were significant differences between the beta diversity of bacterial populations between different regions of the membranes (*p* = 0.052).

The UPGMA dendrogram (Supplementary Fig. 6), generated from the jackknifed PCoA matrices, illustrates the relatedness of the bacterial composition measured in weighted unifrac distances between samples. The unused control membranes (CA, CB, CC) originated from a common node and clustered close to each other. Similarly, mid-feed end communities of clean membranes (14MIDF, 14MIDFb) were closely related to each other and had the longest branching lengths in the dendrogram indicating the bacterial community diversity of these organisms being distinct from others. The dendrogram results support the beta diversity PCoA in Fig. 5.

## Discussion

Fourteen membrane units were autopsied and 16S rRNA gene metabarcoding completed to analyse similarities and differences in the microbial communities. The limiting factor in most biofilm studies is access to a large number of samples from an operational full-scale plant, and duration of their use. It is of importance to study samples that are good models of industrial-scale fouling, in order to better understand the effects of pre-treatment and to design effective methods for prevention and alleviation of biofouling. The present study was novel because of the wide range of membrane samples selected from a full-scale desalination plant, after completion of their functional life-span of 7 years.

### Seawater bacterial community

The bacterial community on RO-membrane biofilms represents a smaller proportion of the bacterial community in seawater. The operational conditions of the plant are likely to favour the selection of a subset of seawater bacteria, which begin their journey from the oligotrophic seawater environment through the pre-filtration steps to carve their niches in the high pressure hyper saline environment on the membranes.

In a closely related pyrosequencing study,^7^ the bacterial community of seawater (Fig. 1), pre-filters and RO membranes from a single unit of the Perth Seawater Desalination Plant were analysed. Many similarities in bacterial families across source water, and membranes were observed, despite disparate locations and seasons. Some of these were *Bacteroidetes (e.g., Flavobacteriaceae), Alphaproteobacteria (e.g., Rhodobacteraceae, Sphingomonadales), Betaproteobacteria* (e.g., *Burkholderia*) and *Gammaproteobacteria* (e.g., *Oceanospirillales*, *Xanthomonadaceae*). There were seasonal variations in the seawater bacterial composition with *Rhodobacteriales* and *Alteromonodales* prevalent during winter and *Enterobacteriaceae* and *Burkholderiales* during summer. The predominant bacterial groups found on RO membranes in the present study were also common to the aforementioned seawater bacterial community, and appear to reflect a source water origin.

There are difficulties in determining the complete range of microorganisms in seawater, because of their very low concentrations. Even triplicate 3 L sample volumes, used in our study may not have been adequately representative of the entire seawater bacterial community diversity. Besides, the filtration of seawater is likely to have artificially selected the organisms that were unable to penetrate a 0.2 µm pore size. Despite the above difficulties, the outcome of the previous study provided valuable qualitative information about the presence of common bacterial groups, particularly the predominant populations such as *Alteromonadales* and *Rhodobacterales* (Fig. 1) strongly suggested a seawater origin of the RO-membrane biofilm bacteria. However, comparative quantification and diversity analysis of the seawater bacterial community and the RO-membrane bacterial community was not possible due to the difference in methods between the two studies.

The coastal waters of southern Western Australia are oligotrophic^8^ in nature with sediments being composed of mainly carbonate sand. The marine water microbes associated with carbonate-rich coastal regions are *Enterobacteriaceae, Pseudomonadales, Rhodobacterales, Caulobacterales, Sphingomonadaceae*, *Burkholderiales and Flavobacterales*.^9^ These bacterial groups were abundant in the membrane biofilm communities, as per our findings, reflecting the ecological influence of surrounding waters.

Cockburn Sound, the feed-water intake site of PSDP, is inhabited by abundant seagrass meadows, a significant reservoir of nitrates.^10^ In Western Australian waters, *Posidonia* genus of seagrass are common; they are known to shed their leaves every 2 months as a survival strategy to prevent surface coverage by calcareous algae. Besides, the periodic shedding of their large pollen grains result in the accumulation of a considerable amount of organic matter in the form of seagrass detritus. In oligotrophic marine environments, seagrass detritus, owing to their low nutrient content are known to form a major source of carbon for denitrifying bacteria, resulting in a high abundance of nitrate-and-nitrite reducers in the seawater.^11^ The above ecological factors are likely to be the reason for a significant proportion of the RO-membrane biofilm community being constituted by nitrate-and-nitrite-reducing bacteria.

### Membrane sampling, biofilm structure, NGS metrics and associated bacterial communities

Membrane fouling is a progressive phenomenon that develops over a period of time, under continuous flowing-water conditions. Initially, the primary colonisers which are transported along the feed-water or endogenously present on the membrane, adhere to the membrane surface. They form the confluent, sticky and irreversibly attached base layer of the biofilm on the membrane surface. As the biofilm matures, secondary colonisers inhabit the membrane surface, adding to the complexity and thickness of the biofilms. The biofilm structure varies between different areas of membranes, depending on how the external factors such as shear force, salt concentration, pressure changes, permeate flux etc. affect the balance between primary and secondary colonisers.

The high numbers of OTUs sequenced from the unused membranes, in relation to their low biomass volume, was unexpected (Fig. 3). The unused membranes were subjected to test runs under flowing water for only short periods, before storage. This may have facilitated the colonisation of membrane surface by water-associated bacteria, and formation of thinner young biofilms made up of mostly bacterial cells and less EPS matrix, contributing to the biomass. Therefore, amplification of DNA from the unused membranes is likely to have recovered a higher number of bacterial OTUs compared to the fouled membranes with a thicker biofilm matrix that possibly interferes with DNA extraction. The large variation in the number of OTUs recovered from the moderately fouled membranes (Fig. 3) was likely due to the presence of thicker (>60 µm) and more heterogeneous biofilms at the feed end from where they were sampled.

The predominant bacterial groups found in our study are known to possess unique physiological properties that are likely to position them favourably in the SWRO environment. *Alpha* and *Gammaproteobacteria*, along with *Sphingobacteriia* are the major primary colonisers, while *Betaproteobacteria* play a significant role as secondary colonisers. *Caulobacterales* was a key group of primary colonisers in our fouled membrane samples (12–16%) and unused membranes to a lesser extent (8%). These bacteria are unique due to their strong prosthecate attachment and alternating motile and sessile developmental stages.^12^ Their stalk cells anchor irreversibly to membrane surfaces via a polysaccharide-based holdfast, also described as the ‘strongest biological adhesion molecule’ or ‘bacterial superglue’^12^ and spread rapidly to form a confluent monolayer or multi-cellular mushroom like structures. The second type of cells known as swarmer cells migrate in search of nutrients and colonise new surfaces.^12^ Compared to most previous studies on desalination systems, our findings differ with respect to the predominance of *Caulobacterales*, suggesting their prevalence in feed-water, characteristic of Western Australian coasts.

Bacteria that produce glycosphingolipids (GSL), namely *Sphingomonadales*, *Rhizobiales* and *Sphingobacteriia* also constituted a substantial portion (>25%) of the biofilm community across all membrane samples, suggesting their crucial role in membrane biofouling. The sticky extracellular biopolymers produced by these groups, e.g., sphingans and gellans protect bacteria from adverse environmental conditions such as extreme pH, temperature, salinity and high pressure.^13–16^ Large amounts of glucuronic acid in the gellans of sphingomonads have a major role in intercellular bridging and also bridging between bacterial cells and membrane surface, thus protecting them from high flow and pressure.^17^ GSL have been only recently identified as important in the biofouling of RO membranes.^17, 18^ They form the base of biofilm matrices,^19^ contribute significantly to the cohesive strength of biofouling layers on RO membranes^20^ and form particularly rigid layers on polyamide surfaces.^18^ GSL and other EPS of sphingomonads have superior attachment efficiency on model polyamide surfaces. They condition RO-membrane surfaces by increasing hydrophobicity.^19^ Our study on the full-scale plant supports recent conclusions about the importance of GSL-producing bacteria in membrane fouling in a laboratory-scale RO desalination system using tertiary-treated wastewater as feed.^21^

Nitrate-and-nitrite-reducing bacteria appeared to play a critical role as secondary colonisers. *Rhizobiales, Burkholderiales* and some members of *Pseudomonadales* are able to reduce nitrates and nitrites, which makes them metabolically versatile under both aerobic and anaerobic conditions.^21^ Of these, *Burkholderiales* migrate in aggregates and form tower-like structures on the top layer of biofilms.^19^ A high abundance of *Burkholderiales* correlated with an increased biofilm thickness in our study and other previous studies.^19^ The co-existence of these groups, alongside strict aerobes such as *Alteromonadales* may help to maintain the ecological balance and maintain optimal substrate utilisation for survival in the harsh SWRO membrane environment.

*Pseudomonas*, a predominant member on both the unused and fouled membranes (11–18%) (Fig. 6, Supplementary Fig. 2) suggested a feed-water origin and resistance to operational conditions. *Pseudomonas* has been recently identified as one of the major RO-membrane biofoulants in a large-scale desalination plant.^22^ Some *Pseudomonas* produce sticky exopolysaccharides like alginate, which favour their attachment to membrane surfaces.^23^ *Pseudomonadales* also migrate in aggregates of bacterial cells clumped together in an EPS matrix, which enables them to persist as secondary colonisers in a mature biofilm.^19^ Many *Pseudomonadales* degrade organic molecules into inorganic substances which may enable them to utilise the metabolic products of initial colonising bacteria. The prevalence on the SWRO membranes described here may reflect their ability to establish and persist as both primary and secondary colonisers. Xanthomonads are known to possess superior attachment capabilities in flowing-water conditionss^24^ owing to the presence of surface structures such as type IV pili^25^ in their outer membranes. The genus *Stenotrophomonas*, which was highly prevalent (10%) on the unused membranes (Supplementary Fig. 2a), is a nitrate-reducer which is an important criterion in biofilm development.^26^

#### Unused membranes

The control unused membranes were invaluable to examine the source of biofouling bacteria and determine whether they originated from upstream feed-water sources or from resident bacteria on the membranes themselves. The fresh polyamide membrane surfaces of the unused membranes can be rapidly conditioned by organic nutrients and polymeric substances. The manufacturing stage of RO membranes does not implement sterile practices. Furthermore, the saline water used for testing is unlikely to be sterile. Hence, it can be expected that *Proteobacteria*, which are ubiquitously found in nature, may be prevalent on the unused and fouled membranes as just inhabitants of the membrane surface initially. The water-associated bacteria, e.g., *Enterobacteriaceae* were also detected on the unused membranes. Operational conditions in the plant may then select only those organisms that survive in the dynamic SWRO environment. To better understand the role of prevalent bacteria in biofouling, we analysed the community composition of fouled and unused membranes with respect to bacterial physiological characteristics and morphological features that may determine their relative abundance in various membranes.

The predominance of primary colonisers such as *Alpha* and *Gammaproteobacteria* (Fig. 6) that possess superior attachment and adhesion capabilities,^27^ on the unused membranes was reflected by their low biofilm thickness (~30 µm) and biomass volume, in comparison to that of the fouled membranes. This indicated the presence of younger, thin biofilms on the unused membrane surfaces.

Prevalent orders among the *Gammaproteobacteria* on unused membranes were *Pseudomonadales* and *Xanthomonadales*, the fouling properties of which have been described above. It was an interesting finding that *Escherichia/Shigella* were present at 5% abundance (Supplementary Fig. 2a) on the unused membranes. These are likely to have originated from either the surface water used for testing or contamination during manufacture. In our preliminary pyrosequencing study,^7^ a high abundance of *Enterobacteriaceae*, which included *Escherichia*, was recorded in cartridge filter samples, perhaps suggesting a feed-water origin. Seawater has been suggested as the relevant reservoir of *E.coli* strains adapted to marine environments.^28^

There is no continuous water flow to bring about the deposition of further nutrients and bacteria on the unused membranes. The conditions of limited oxygen and organic carbon sources, that occur on unused membranes, are reflected by a high abundance of the GSL-producing and nitrate-and-nitrite-reducing *Rhizobiales* (24%), particularly members of the genus *Ochrobactrum* (21%).

The least prevalent class on the unused membranes were *Betaproteobacteria*, mainly *Burkholderiales*. These are usually considered to be secondary colonisers but in this study were evident before operational use. It was an important observation that the relative abundance of the nitrate-and-nitrite-reducing bacterial groups was the highest on unused membranes (>50%), reflecting the limited nutrient availability and anaerobic conditions on the unused membranes within the sealed units, before operational use.

The unused control membranes hosted bacterial communities that were not only significantly different from those of fouled membranes but also the least diverse, due to the predominance of only *Proteobacteria*. The fouled-membrane environment selected for bacteria that were resistant to operational conditions, hence a higher diversity was observed. There was a significant difference in species richness between severely fouled and unused membranes.

#### Fouled membranes

Bacterial communities on fouled membranes represented stable, mature biofilms formed over a period of 6 to 7 years under continuous high velocity of water flow, high pressure and salinity. These conditions are likely to select specific populations able to withstand these adverse conditions and reach a balanced and coordinated multi-species community. Compared to unused membranes, the proteobacterial abundance was lower and the *Bacteriodetes* (mostly *Sphingobacteriia* and *Flavobacteriia*) abundance was higher per unit of biomass volume. This corresponds with a drop in strictly anaerobic bacteria and an increase in the proportion of GSL producers which may be more halophilic. The gliding motility of *Sphingobacteriia* and *Flavobacteriia*^29^ enables them to move and colonise the rough surface of membranes, position themselves and proliferate at optimal levels of oxygen, carbon source, temperature, light intensity, salinity, pressure and shear force resulting in their high prevalence on the fouled membrane biofilms. The SWRO membrane surface may have favoured the selection of GSL-producing bacteria due to the constant nutrient supply of adhered polymers. The high pressure and flow rate may also facilitate the fast-spreading nature of *Sphingomonadales* in a monolayer, providing larger surface contact area and hence better access to nutrients which accumulate at interfaces such as the membrane surface and from neighbouring bacteria. The fouled membranes harboured *Rhizobiales*, perhaps due to their superior ability to survive under anoxic conditions. There was also an increase in the relative abundance of *Caulobacteriales* on fouled membranes (7–18%) compared to the unused membrane biofilms (7.7%) per unit of biomass volume. As loosely attached bacteria would tend to be removed from the membrane surface by the high flow/shear, primary colonisers must be permanently and irreversibly attached to the membrane surface. The holdfast of *Caulobacteriales*, may have enabled their stalk cells to anchor themselves permanently on membranes while swarmer cells may have migrated along flowing seawater and proliferated rapidly to inhabit fresh surfaces, thus increasing their abundance.

*Betaproteobacteria*, comprising of mostly *Burkholderiales*, were also more relatively abundant on the fouled membranes (up to 30 %) compared to unused membranes (24%). *Burkholderiales*, previously described as a major component of RO-membrane biofilms^30^ are known to migrate as free-floating aggregates of bacterial cells embedded in their EPS matrix^31^ and possibly originate from detached portions of biofilms from upstream locations such as cartridge filters or water pipes.

On the clean membranes, the biofilm thickness was close to that of severely fouled membranes (~ 60 µm). Conditions on clean membranes selected for primary colonisers due to insufficient nutrients to support secondary colonisers, or by failure of secondary colonisers to efficiently reach and colonise the surface. The higher relative abundance of *Caulobacterales* and GSL-producing bacteria together (45%) on the clean membranes may be due to their superior attachment capabilities and production of large amounts of EPS which enable them to survive the stress of shear force, high salinity, pressure and nutrient depletion. Clean membranes may have been exposed to higher shear forces leading to washing away of loosely attached secondary colonisers resulting in less compact biofilms with low biomass volume (Fig. 2), but of similar thickness as severely fouled membranes. *Burkholderiales*, being one of the most abundant orders on clean membranes (Fig. 6b), suggested the presence of secondary colonisers as part of loosely attached or partially dispersed biofilms.

The moderately-fouled membranes hosted a diverse, species-rich and stable bacterial community (Fig. 6c), with fairly uniform abundance of all major taxonomic orders. Higher diversity can increase resistance of the population to stress and lead to greater biofilm stability due to differences in physiology and increased co-metabolism options.^32^ Despite a higher biomass volume, the moderately-fouled membranes had a lower biofilm depth (~50 µm) than that of the clean membranes, indicating that the greater compactness of the biofilm on moderately-fouled membranes was a result of exposure to high shear rate. Predominance of *Xanthomonadales*, *Sphingomonadales* and *Caulobacterales* (Fig. 6c) suggested that the high shear force may select for bacteria with superior attachment ability and those that produce very sticky polysaccharides that bring about a decline in permeate flux to counteract the shear force. Previous research shows that *Xanthomonadales* are an integral part of membrane biofilms subjected to high shear rate^33^ and exhibit significantly higher attachment than other bacteria in mixed cultures.^24^

A higher alpha diversity is an indicator of higher stability of biofilms, therefore, it may be suggested that the moderately fouled membranes hosted the most versatile biofilms (Table 2). However the beta diversity was close to those of clean and severely fouled biofilms (Fig. 5b), indicating that variation in beta diversities among fouled membranes was due to the difference in relative abundance of the same genera rather than absence or presence of specific genera.

The thicker biofilms (>64 µm) on severely fouled membranes indicated a stable, mature biofilm and the bacterial community data supported this. The severely fouled membrane biofilms (Fig. 6d) were predominated by *Burkholderiales*, which were possibly detached from upstream biofilms and deposited on the preformed primary layers, thus increasing the biofilm thickness.^19^ The stickiness and adhesion to the primary colonisers’ EPS layer may then prevent these secondary colonisers from migrating to new surfaces. Furthermore, the denitrifying capabilities of these bacteria enable them to survive for long periods on organic decomposition under limited nutrient and oxygen supply.

As conditions change in different locations within a SWRO membrane unit, irrespective of how severely fouled it is, we also compared communities from different locations of both clean and severely fouled units pooled together. The feed and middle regions of membranes showed the most similar community composition (Fig. 7), perhaps due to similar conditions of nutrient and oxygen availability, salt concentrations and progressively decreasing permeate flux. The biofilm thickness gradually decreased from the feed end (>60 µm) towards the middle (>45 µm) and this was supported by the NGS results which showed a lower relative abundance of *Burkholderiales* in the middle region of membranes compared to the feed end (Fig. 7).

The feed end of membranes (Fig. 7a), where pre-filtered seawater first enters the SWRO unit, carries the highest levels of nutrients and oxygen but also highest levels of potential foulants such as free-floating loose polysaccharides, proteins or bacteria. These conditions may facilitate build up of thicker biofilms as is reflected by the high relative abundance of *Burkholderiales*, compared to other locations. The stickiness of the GSL producers and *Caulobacterales* in the primary layer prevents these secondary colonisers from detachment and migration to other locations. It may therefore be suggested that the feed end of membranes have a more stable and mature biofilm with strongly adhesive primary colonisers than the other membrane locations.

Due to their spiral-wound format, the mid-feed location of SWRO membranes (Fig. 7b) would be expected to have similar conditions as those of feed end, but may have higher dissolved salts and a continuous supply of oxygen. It may also be assumed that the highest shear force and hence maximum permeate flux could be expected in the mid-feed end locations. These conditions may select for organisms that are strictly aerobic and are only able to survive in the presence of high concentrations of sodium ions. The mid-feed biofilms may have favoured the selection of *Alteromonadales*, which are strict aerobes, and halophiles over *Xanthomonadales* as primary colonisers resulting in a shift in the gammaproteobacterial composition (Fig. 7b).

*Pseudomonadales* and *Alteromonadales*, which were dominant in the feed-water and polished seawater, respectively (Fig. 1), were also relatively more abundant in the mid-feed and reject locations (Fig. 7b, d), which have the highest exposure to seawater compared to other regions of the spirally-wound membranes. The above findings largely support the fact that seawater bacterial community directly or indirectly influences the ecological structure of the membrane bacterial community. Although the high shear force may select for organisms that can adhere permanently to membranes with cohesive EPS, the strictly aerobic conditions may enable only a few of these groups to survive. This may be the reason for a comparatively lower abundance of GSL-producing bacteria and the observed shift to aerobic *Sphingobacteriia* over facultatively anaerobic *Sphingomonadales* and *Rhizobiales*. The secondary colonisers such as *Burkholderiales* may attach to the primary fouling layer initially but it is possible that they might have been easily detached from the surface layers, partly due to the inability of EPS in primary layer to hold them strongly, and partly due to their reduced survival under high oxygen concentrations. Most species of *Flavobacteriia* are aerobic, hence they may be prevalent in higher abundance in these locations.

Compared to the mid-feed end, the middle region of the membranes may have a lower flow rate and lower permeate flux caused by resistance of the fouling layer as feed-water flows along the membrane, making transportation of detached bacteria and nutrients more difficult. The membrane surface in these areas may therefore, be oligotrophic. The limited supply of nutrients and oxygen downstream supports the survival of metabolically adaptive secondary colonisers such as *Betaproteobacteria* to a greater extent than the mid-feed location. This may suggest why there was a slightly higher abundance of *Alpha* and *Betaproteobacteria* in the middle (Fig. 7c), compared to the mid-feed locations.

At the reject end (Fig. 7d), there was a higher relative abundance of *Sphingobacteriia* and GSL-producing microbes. Sphingomonads may have survived the various stress factors better than other microbes due to their close and strong association with the membrane surface. They are known to effectively colonise RO membranes in continuous flow systems, regardless of the surface properties of the membranes.^34^ Their metabolic adaptability to a wide range of both natural organic sources and environmental contaminants^35^ enables them to survive even high nutrient concentrations on the membrane surface due to a concentration polarisation effect and accumulation of nutrients in the biofilm matrix.^36^ The above factors place sphingomonads in a superior position for retention/accumulation in mature SWRO membrane biofilms from the feed to the reject end.

In summary, bacterial communities in clean and severely fouled membranes differed in the prevalent orders of bacteria, while moderately fouled membranes hosted a more diverse population indicating their versatility. The feed end biofilms were the most mature and stable, with a high abundance of *Betaproteobacteria*. Biofilms in the mid-feed were the most compact and selected for strict aerobes, the middle region biofilms were well adapted to oligotrophic conditions with more or less similar proportions of *Alpha, Beta, Gammaproteobacteria* and Sphingobacteriia. The reject end biofilms were loosely bound, less compact biofilms with a positive selection for *Alphaproteobacteria* and GSL-producing bacteria that may have sustained the shear forces.

Considering the above factors, in order to overcome the bias of flow conditions at the feed end in favour of certain groups of bacteria, it is important to sample from various locations along membrane sheets as they represent differences in bacterial communities in full-scale plants along the spirally-wound unit.

## Conclusion

The findings of the present study were novel because of the wide range of membrane samples from a full-scale desalination plant, after completion of their functional life-span. Control unused membranes provided a strong basis of analysing whether biofouling bacteria originated from upstream feed-water sources or from resident bacteria on membranes themselves. It may be beneficial to minimise membrane contamination and dormant bacterial load, especially primary colonisers such as *Caulobacterales* inhabiting unused membranes prior to use.

Variations in communities with differing severity of fouling and membrane location were mainly based on ecological balance between primary colonisers with strong attachment mechanisms (e.g., *Caulobacterales, Pseudomonadales, Xanthomonadales*), glycosphingolipid-producing bacteria (*Sphingomonadales, Rhizobiales, Sphingobacteriia*) and secondary colonisers with denitrifying properties such as *Burkholderiales*. Moderately fouled membrane communities were the most diverse and those on mid-feed end of membranes, most different from other fouled membranes.

Our data support that the prevalence of bacterial groups containing sphingolipids significantly contribute to fouling, and treatments to target and disrupt them may offer improved removal of SWRO membrane biofilms. The importance of the holdfast mechanism in *Caulobacterales*, which were found to be permanent inhabitants on all sampled membrane surfaces is another area to be further investigated.

Polysaccharides of holdfast and GSL may be expected to form some of the strongest structures of the entire biofilm matrix. Future research and control methods may be directed towards further investigation on targeting these compounds specifically to alleviate fouling. In a further study, we characterised exopolysaccharides from model bacterial strains isolated from the same plant and it was interesting to detect similarities to glycosphingolipid composition, in all our EPS samples from non-GSL-producers.^37^ Apart from the intrinsic factors on the membrane surface other factors that may influence the ecological balance are microbial community in feed-water, seasonal and temporal variations in feed-water composition, and also effect of periodic pre-treatment and cleaning methods used in the plant. Further sequencing studies could include feed-water and pre-filter locations for a more detailed comparison.

The bacterial community in seawater was artificially selected by filtration; therefore, the possibility of NGS analysis to be biased in favour of organisms that are unable to penetrate a pore size of 0.2 µm, cannot be ruled out. A few membranes had large proportions of particular species, this cautions against relying on sampling too few membranes, which could be misleading in interpreting their true importance within the communities. For future studies, we suggest sampling at least five membranes from each of the locations of the SWRO membrane unit as described in this study. Samples may also be included at periodic time intervals to study the seasonal variations in bacterial community structure.

## Methods

### Preliminary seawater sample analysis

As part of a related preliminary study,^7^ triplicate samples of source feed-water and polished seawater (post-RO-filtration stage) were collected in 3 L volume each, and concentrated by vacuum filtration through 0.2 µm polycarbonate filters. Each filter was then cut into smaller pieces for DNA extraction using a MoBio (Solana Beach, CA) Powersoil DNA kit, following the manufacturer’s instructions, with final DNA elution carried out in 30 µL volumes. The DNA was subjected to PCR amplification of the V1-V2 hypervariable region of the bacterial 16S rRNA gene, by barcoded pyrosequencing as previously described.^38^ Briefly, universal bacterial fusion primers 27F (5′-AGAGTTTGATCCTGGCTCAG-3′) and 338R (5′-CATGCTGCCTCCCGTAGGAGT-3′)^38^ were used to generate PCR amplicons in triplicate, and pooled. PCR was carried out in a 25 µL total volume including 4 µL of template DNA, containing: 2.5 mM MgCl2 (Fisher Biotec, Aus), 1 × Taq polymerase buffer (Fisher Biotec, Australia), 0.4 µM dNTPs (Astral Scientific, Australia), 0.4 mg BSA (Fisher Biotec, Australia), 0.4 µM of each primer, and 0.25 µL of AmpliTaq Gold DNA polymerase (ABI). The PCR conditions included: initial denaturation at 95 °C for 5 min, followed by 40 cycles of 95 °C for 30 s, 54 °C 30 s, 72 °C for 30 s, and a final extension at 72 °C for 10 min (Corbett Research, NSW, Aus) on a PCR thermal cycler (Bio-Rad). Amplicons were purified (AMPure beads, Invitrogen) and DNA concentration estimated by ethidium gel staining to approximate equimolar concentrations for emulsion PCR. Bead: template rations for the emulsion were determined by qPCR. The Roche GS Junior run set up included an emulsion PCR step, bead recovery, and the sequencing run. All of these procedures were carried out according to the Roche GS Junior protocols (http://www.454.com). The sequencing output files were processed as previously described^39^ through an automated pipeline in an Internet-based bioinformatics workflow environment, YABI (https://ccg.murdoch.edu.au/yabi/). The resultant BLAST files were imported into the MEGAN4 MEtaGenome ANalyzer (version 4.62.1)^40^ for taxonomy.

### SWRO membrane sampling

Reverse osmosis membranes were obtained from the Perth Seawater Desalination Plant, Western Australia, after they had been in continual use for ~7 years. The SWRO membranes were replaced due to a marked drop in permeate flux, and increased energy usage in the plant. The SWRO design comprises of a first pass with 12 trains fitted with 162 pressure vessels each; and a second pass with 6 trains, fitted with 12 pressure vessels each (Supplementary Fig. Supplementary Fig. 7). Autopsy of SWRO membranes was performed across various labelled individual spirally-wound units (*n * = 14) to include a diverse range of samples. Each unit consisted of up to 30 leafs of polyamide membranes (Dow Filmtec). Each leaf was made of two membrane sheets measuring 20 × 100 cm, glued together back-to-back with a permeate spacer between them.

The membrane samples were categorised according to both their fouling severity [clean (*n* = 5), moderately fouled (*n* = 2), severely fouled (*n* = 4)] and location [feed end (*n * = 2), mid-feed (*n * = 3), middle (*n* = 2), or reject end (*n * = 2)]. One unused (CA,CB,CC) and one severely fouled membrane (9A,9B,9C) was sampled and extracted in triplicate to check that each sample was a good representative of larger areas of membranes that belonged to the respective category. The sampling classification is presented in Table 1.

### Confocal laser scanning microscopy (CSLM) and biomass measurements

Microscopy of membranes was performed to analyse the biofilms as described previously.^41^ Membrane samples of the control group and each of the groups of varying fouling severity, were cut into coupons of approximately 1 cm^2^ area, in at least triplicate, and stained with FilmTracer^™^ LIVE/DEAD biofilm stain (Invitrogen), according to the manufacturer’s instructions. The membranes were wet mounted on clean glass slides, secured with cover slips and observed on a Nikon C2 CLSM microscope at 20 × magnification, using associated software (NIS-Elements) to collect z-stacks of at least three areas per membrane. Imaris (Bitplane version 8.4.1) software was used to reconstruct the 3D images of these three areas for quantification of membrane biofilms. Average values of biomass volume and biofilm depth were calculated from the images. The biovolume is defined as the number of biomass pixels in all images of a stack multiplied by the voxel size [(pixel size)_*x*_ × (pixel size)_*y*_ × (pixel size)_*z*_] and divided by the substratum area of the image stack. The resulting value is the biomass volume divided by substratum area (μm^3^/μm^2^), expressed in μm^3^ units (Imaris). Biovolume represents the overall volume of the biofilm, and also provides an estimate of the biomass in the biofilm.^42^ The average biomass volume of the unused membranes was normalised to a value of one, for the purpose of comparison against the biomass volume of fouled membranes and for calculation of abundance of taxa per unit biomass volume of the corresponding membrane groups.

### DNA extraction

Initially, DNA was extracted from membrane samples using the PowerSoil^®^ DNA Isolation Kit (MO BIO, Qiagen) for soil and environmental samples, as per the manufacturers’ instructions. However, on quantification of DNA, this method resulted in low yields of poor quality DNA from 0 to 8 ng/μL. Therefore, genomic DNA was isolated from 0.5 g samples of the SWRO membranes following the protocols of Urukawa and colleagues.^43^ Briefly, 0.5 g of SWRO membranes were pulverised by bead beating in a Lysing Matrix E tube (MP Biomedicals), then mixed with 350 μL phenolchloroform-isoamyl alcohol (50:49:1) (TE saturated; pH 8.0) and 350 μL 2xTENS buffer, and centrifuged at 16,000 × *g* for 5 min. The aqueous phase was aspirated into a 2 mL Phase Lock Gel tube (Sigma Aldrich), gently mixed with 300 μL 7.5 M ammonium acetate, and an equal volume of chloroform, and centrifuged at 16,000 × * g* for 5 min. DNA was recovered from the phenol-chloroform extraction by ethanol precipitation with 0.6 × volumes of ice-cold isopropanol and 3 μL of GlycoBlue (Ambion, Austin, USA), following standard protocols, and resuspended in 50 μL TE buffer. DNA extractions were performed in duplicate for each samples, and DNA yield and purity was assessed using a Nanodrop spectrophotometer (Thermo Scientific); up to 150 ng/μL of DNA was isolated per sample (Table 1). Extraction reagent blank controls were performed alongside all DNA extractions.

### Bacterial 16S rRNA gene amplicon library preparation and MiSeq sequencing

The V4 hypervariable region of bacterial 16S rRNA genes in the SWRO membrane DNA samples were amplified by PCR using the 515F (5′-GTGCCAGCMGCCGCGGTAA-3′) and 806R (5′-GGACTACHVGGGTWTCTAAT-3′) primers described by Caporaso and colleagues.^44^ PCRs were performed in 25 μL volumes containing 1 × PCR buffer, 2.5 mM MgCl_2_, 1 mM dNTPs, 400 nM of each primer, 1U PerfectTaq polymerase (5 PRIME, Germany), and 2 μL of DNA. No-template and extraction reagent blank controls were included in all PCR runs. PCR thermal cycling conditions were: 95 °C for 5 min followed by 34 cycles of 95 °C 30 s, 55 °C for 30 s and 72 °C for 30 s, and a final extension of 72 °C for 5 min in a Bio-Rad Thermal Cycler. PCR amplicons were electrophoresed though 2% agarose gels stained with GelRed (Fisher Biotech) and visualised under UV light). From the resulting bacterial 16S rRNA gene amplicons, sequencing libraries for the MiSeq sequencing platform were produced according to Illumina recommended protocols (Illumina Demonstrated Protocol: 16S Metagenomic Sequencing Library Preparation), with amendments. Purified, uniquely indexed libraries from individual DNA samples were pooled for sequencing in equimolar quantities based on the fluorescent intensity of amplicon libraries after electrophoresis though a 2% agarose gel stained with GelRed (Fisher Biotech) and visualised under UV light. Sequencing was performed on an Illumina MiSeq using 500-cycle V2 chemistry (250 paired-end reads) following the manufacturer’s recommendations.

### Next generation sequencing analysis

Sequences were first subjected to quality control (QC) procedures as previously described.^45^ Briefly, paired-end reads were merged using USEARCH v8^46^ with a minimum overlap length of 50 bp and no gaps allowed in the merged alignments. Primer sequences and distal bases were trimmed from the ends of reads in Geneious v8.1.6 (Biomatters, New Zealand)^47^ and sequences of less than minimum reported size for the 515F/806R amplicons (200 bp) were removed. Singleton sequences (per sample), and sequences with a >1% error rate were removed from the dataset using USEARCH v8.^46^ Operational taxonomic units (OTUs) were created by clustering sequences at 97% similarity and 2456 OTUs were clustered using the UPARSE algorithm.^48^ Of these, 5% were chimeric and were removed with the UCHIME algorithm.^49^ Taxonomy was assigned to OTUs in QIIME^50^ by aligning to the GreenGenes 16S rRNA database^51^ using the UCLUST algorithm^46^ with default parameters. OTUs taxonomically assigned to the class, family or genus-level were used for further analysis. The number of OTUs sequenced for each membrane sample were analysed against the respective biomass volume of the membrane biofilms. OTUs present in control samples (PCR no-template controls and DNA extraction reagent blank control) were removed from the dataset to remove all background sequences present in the environment and through handling of samples. Following this process, a total of 343,000 unique sequences were represented by 2229 OTUs. To compare the bacterial community structure between replicate samples of the control groups, and between different membrane classifications, the abundance of the major taxonomic groups was calculated as a proportion per unit biomass volume of the corresponding membranes.

Bacterial diversity estimates and statistical analyses were performed in QIIME v1.9.0.^52^ Estimates of bacterial alpha diversity were calculated after multiple rarefactions to a maximum depth of 50,507 sequences using the Choa1^53^ and Shannon diversity indices.^54^ Statistical comparison of bacterial diversity levels between SWRO membrane treatment groups was calculated on rarefied data (3254 sequences per sample) with parametric pair-wise t-tests. Beta diversity estimates were calculated on a rarefied subsample (3254 sequences per sample) with jackknifed PCoA using weighted Unifrac distances,^55^ and a UPGMA dendrogram^56^ was generated in QIIME from the jackknifed PCoA matrices. PERMANOVA tests were used to assess the statistical differences between SWRO bacterial communities.^57^

### Data availability

The datasets generated during the current study are available in the Figshare repository, https://doi.org/10.6084/m9.figshare.4955708.v157.

## Electronic supplementary material

Supplementary Figure 1

Supplementary Figure 2a

Supplementary Figure 2b

Supplementary Figure 2c

Supplementary Figure 2d

Supplementary Figure 3

Supplementary Figure 4

Supplementary Figure 5

Supplementary Figure 6

Supplementary Figure 7

Supplementary Table 1a

Supplementary Table 1b

## Electronic supplementary material

**Supplementary Information** accompanies the paper on the *npj Biofilms and Microbiomes* website (doi:10.1038/s41522-017-0021-6).
