# Supplementary figures and images for: Characterisation and comparison of bacterial communities on reverse osmosis membranes of a full-scale desalination plant by bacterial 16S rRNA gene metabarcoding
Source: NPJ Biofilms Microbiomes. 2017 Jun 19;3:13. doi: 10.1038/s41522-017-0021-6 (PMC5476683; doi:10.1038/s41522-017-0021-6)

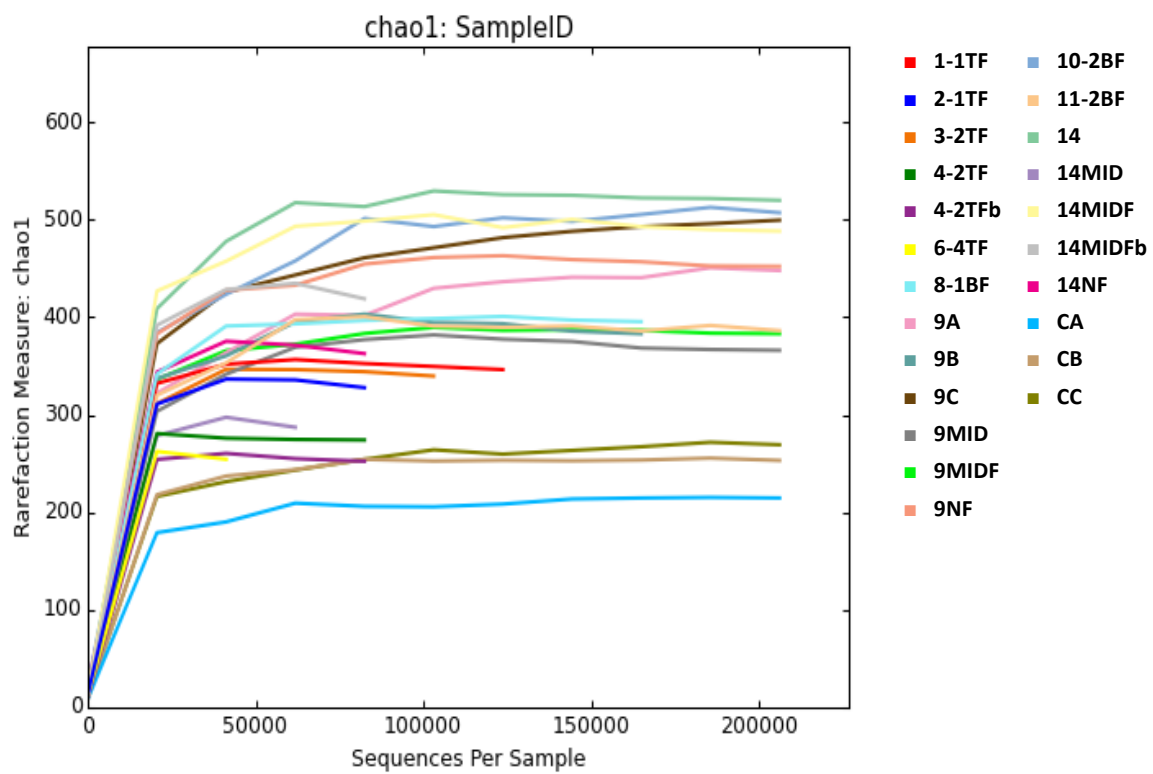

Supplement: Supplementary file 1 — Supplementary Figure 1 [file 41522_2017_21_MOESM1_ESM.pdf]

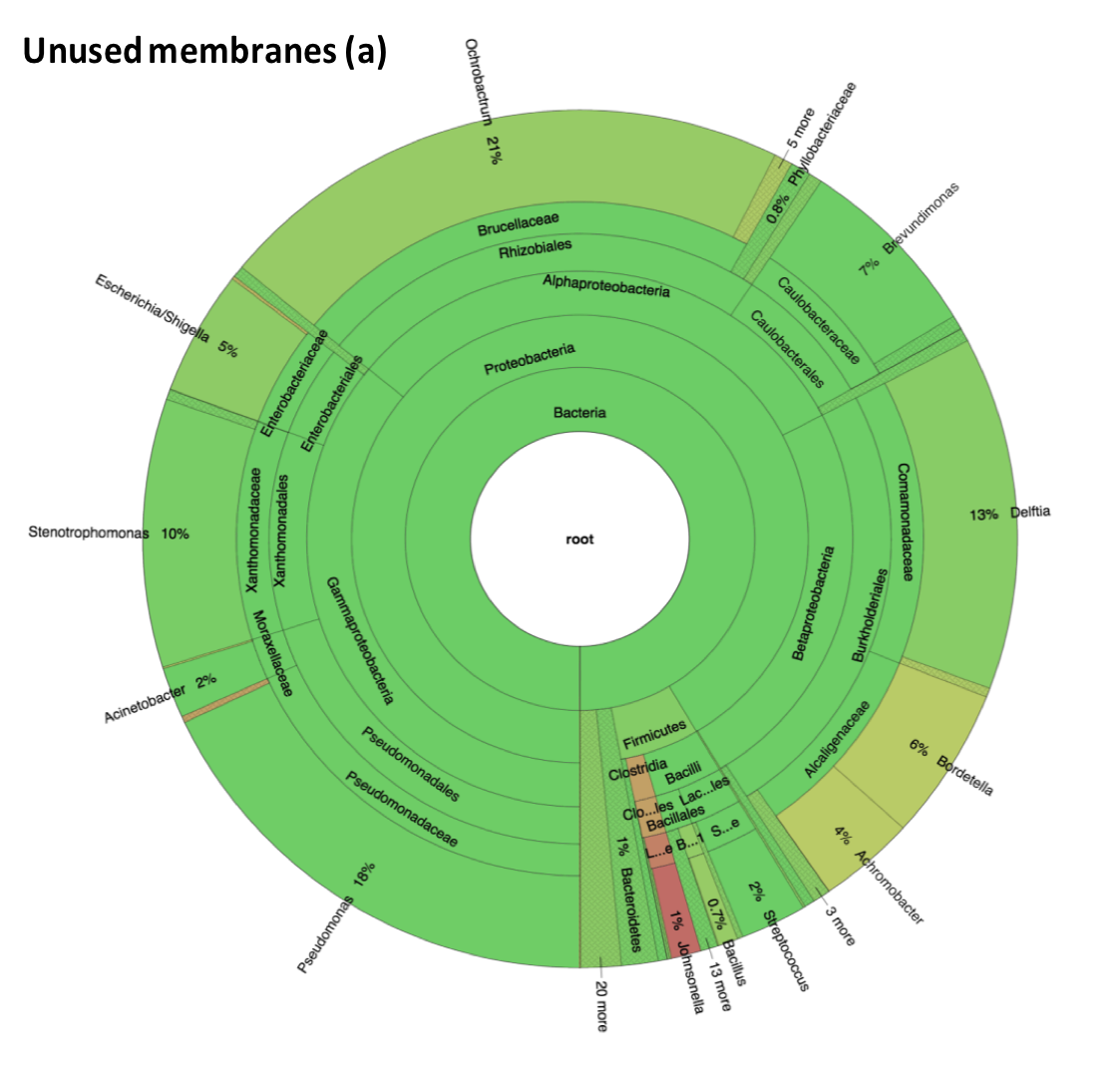

Supplement: Supplementary file 2 — Supplementary Figure 2a [file 41522_2017_21_MOESM2_ESM.tif]

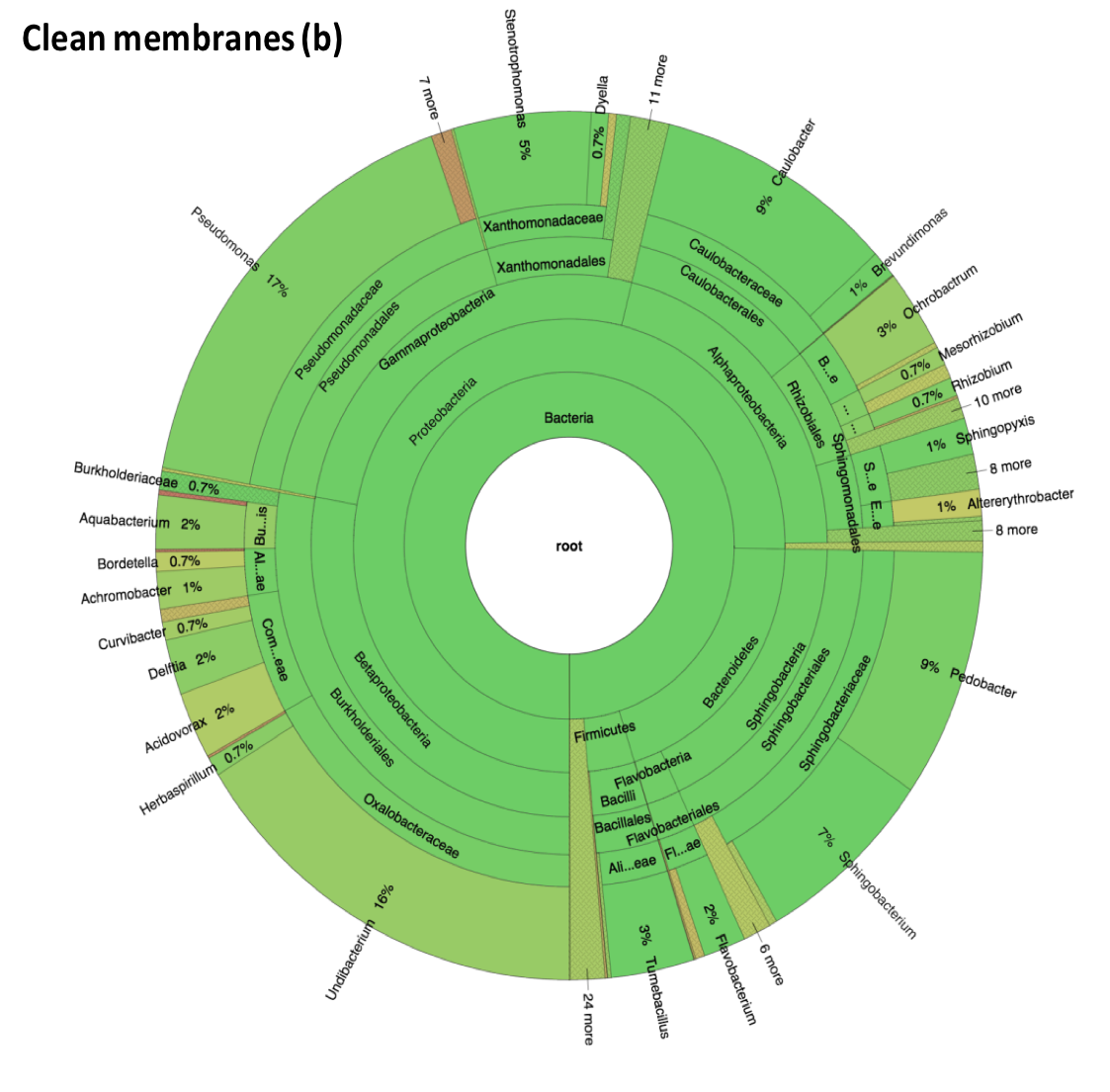

Supplement: Supplementary file 3 — Supplementary Figure 2b [file 41522_2017_21_MOESM3_ESM.tif]

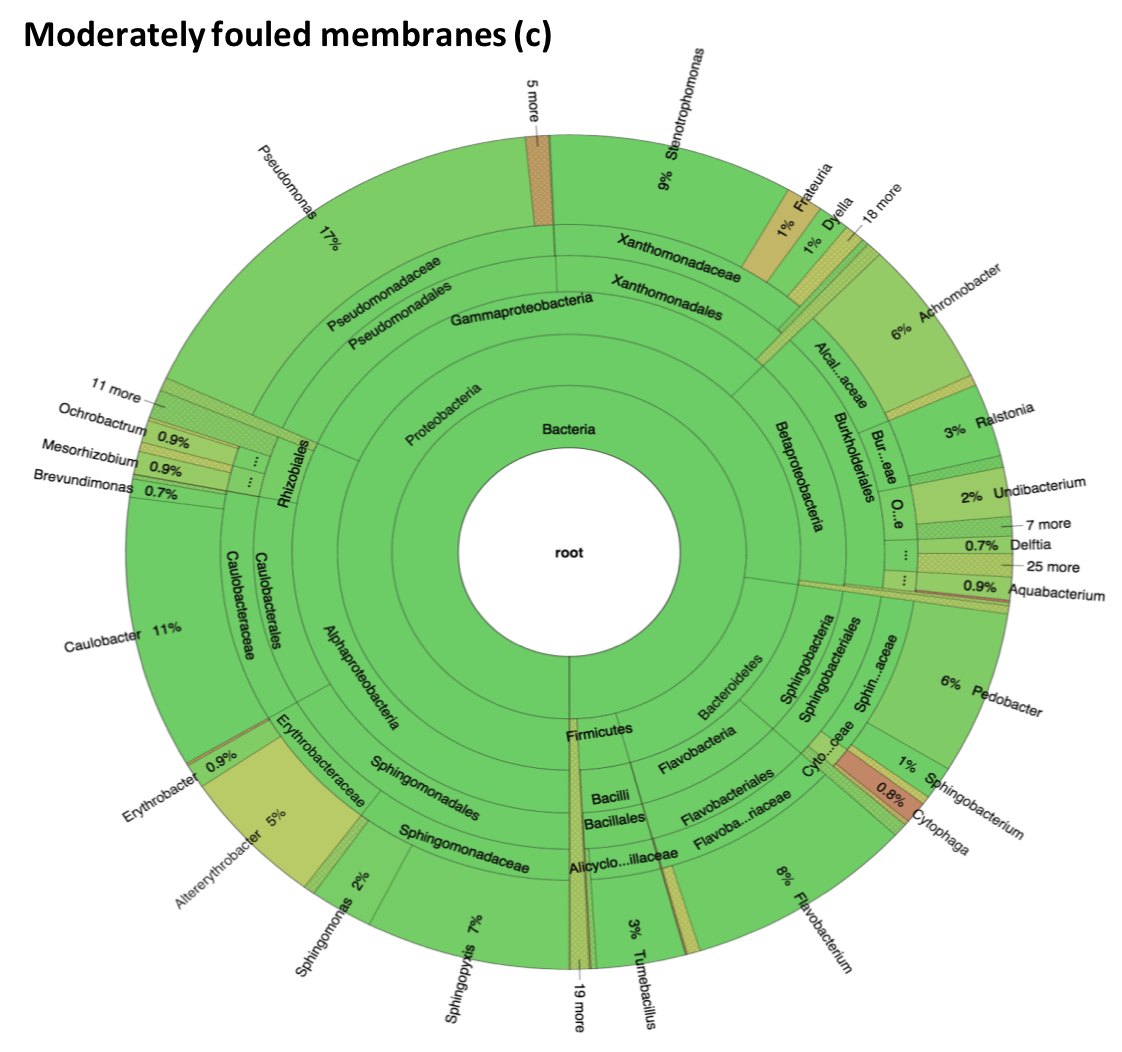

Supplement: Supplementary file 4 — Supplementary Figure 2c [file 41522_2017_21_MOESM4_ESM.tif]

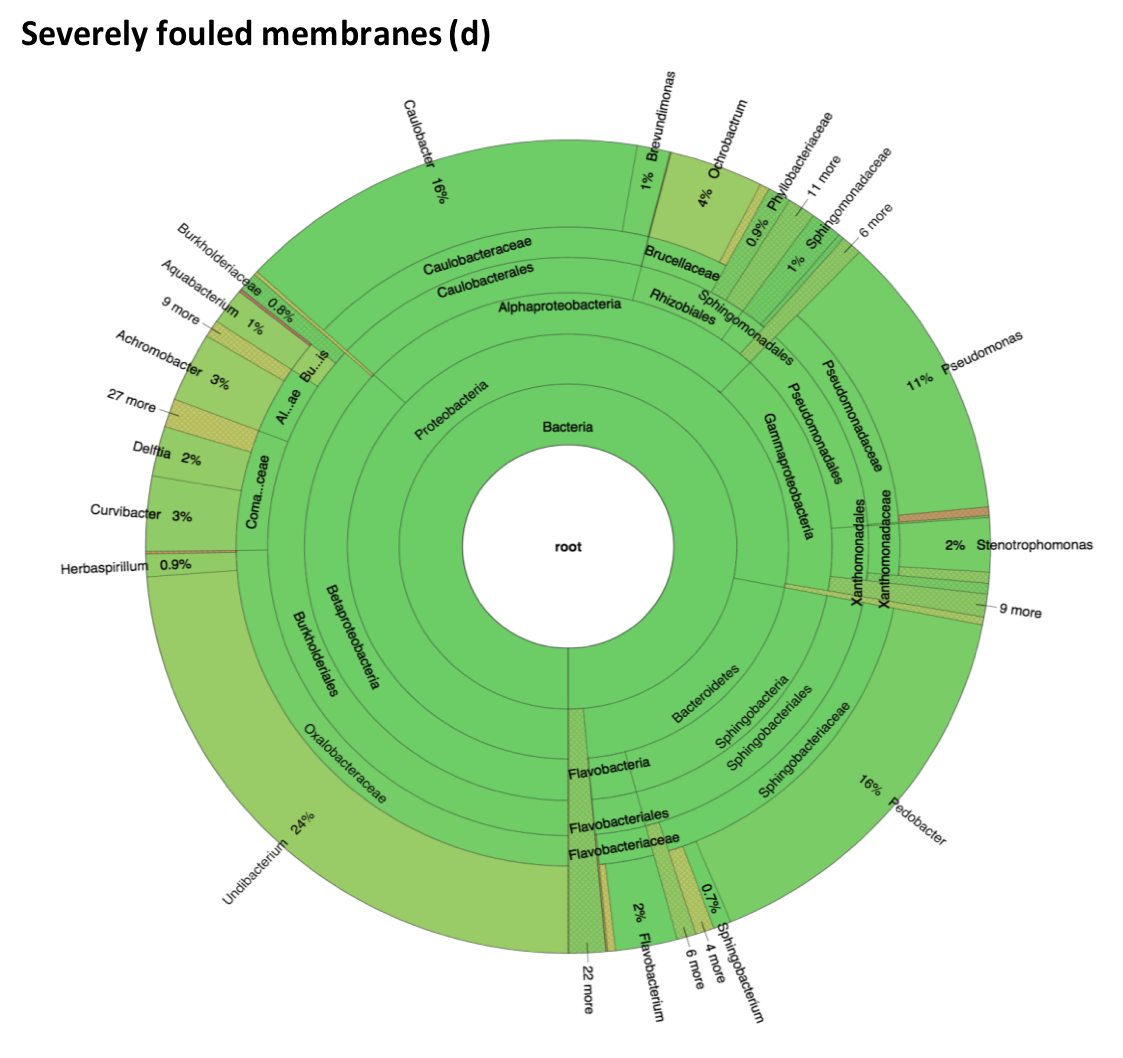

Supplement: Supplementary file 5 — Supplementary Figure 2d [file 41522_2017_21_MOESM5_ESM.tif]

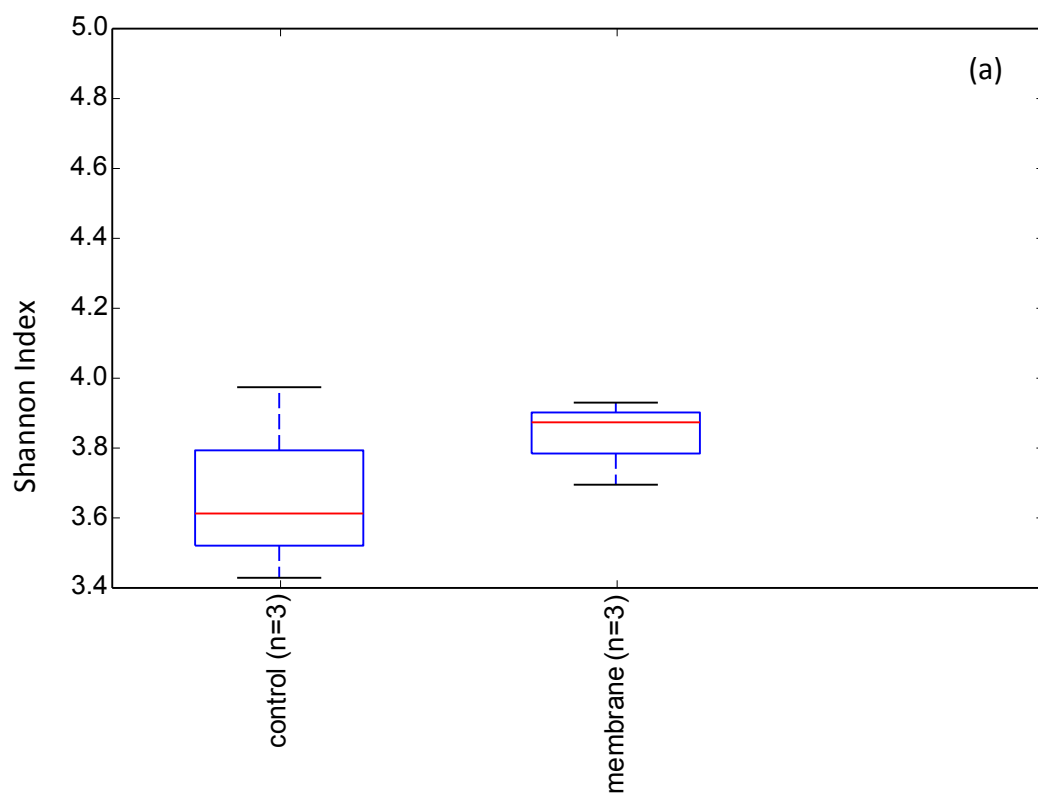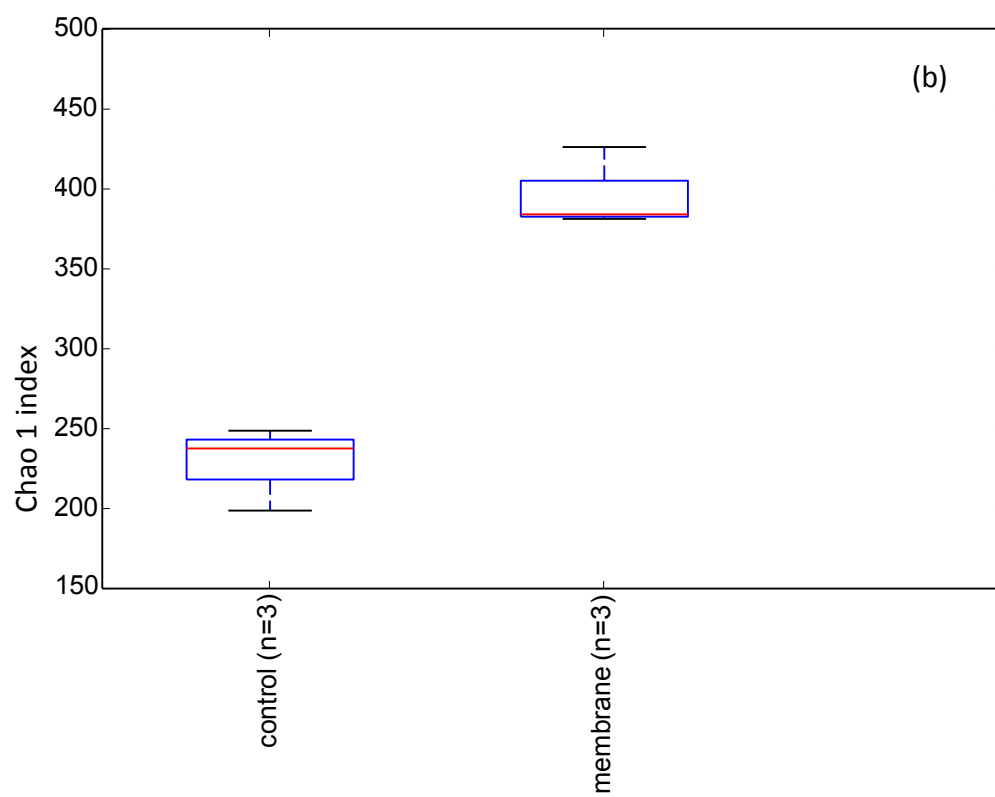

Supplement: Supplementary file 6 — Supplementary Figure 3 [file 41522_2017_21_MOESM6_ESM.pdf]

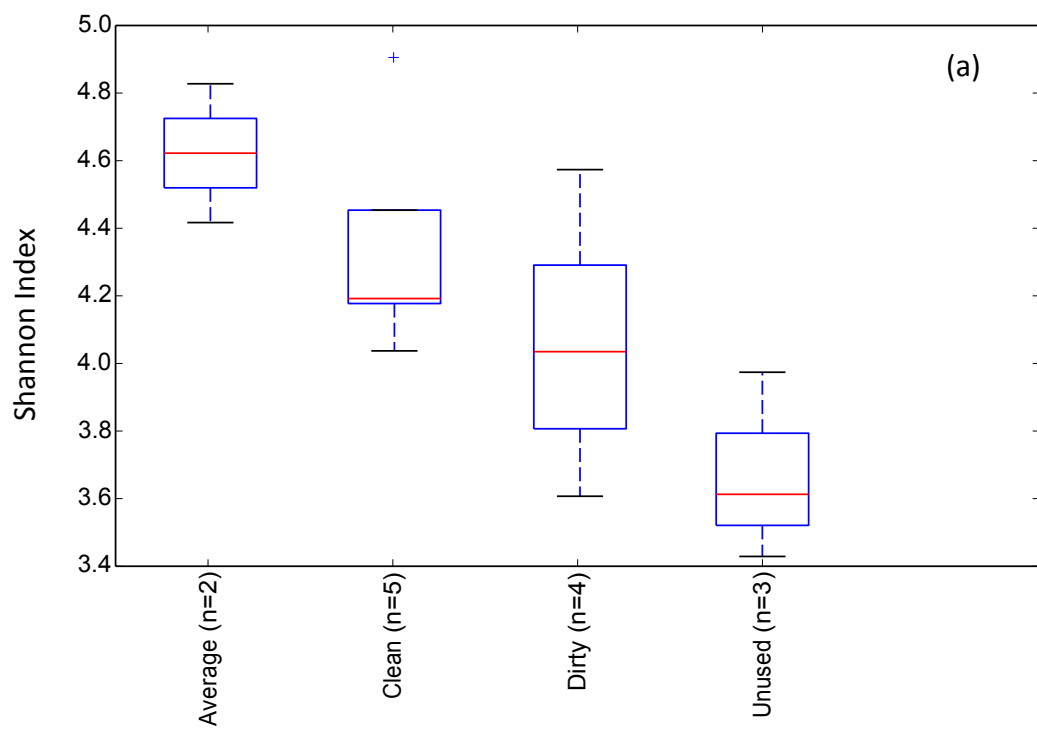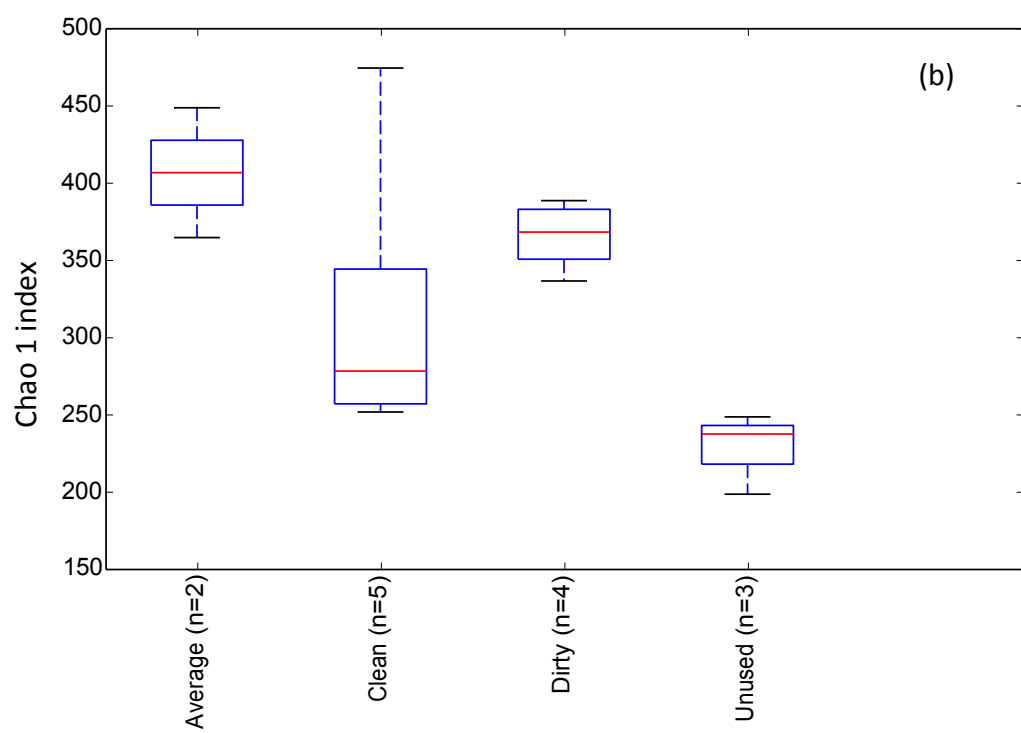

Supplement: Supplementary file 7 — Supplementary Figure 4 [file 41522_2017_21_MOESM7_ESM.pdf]

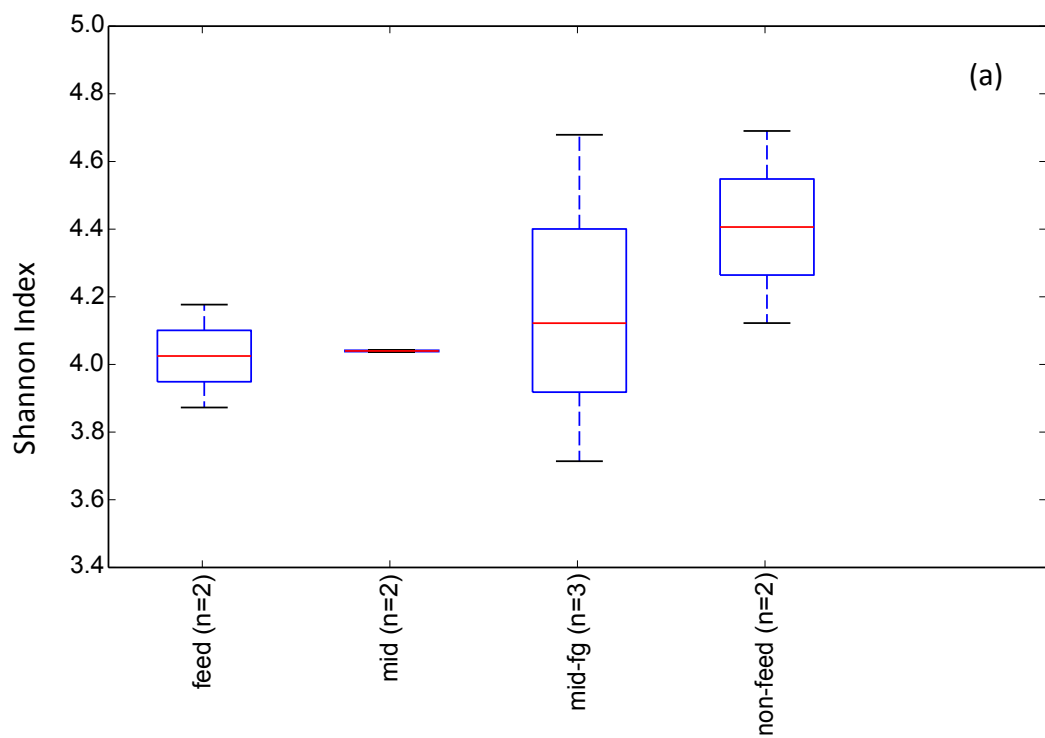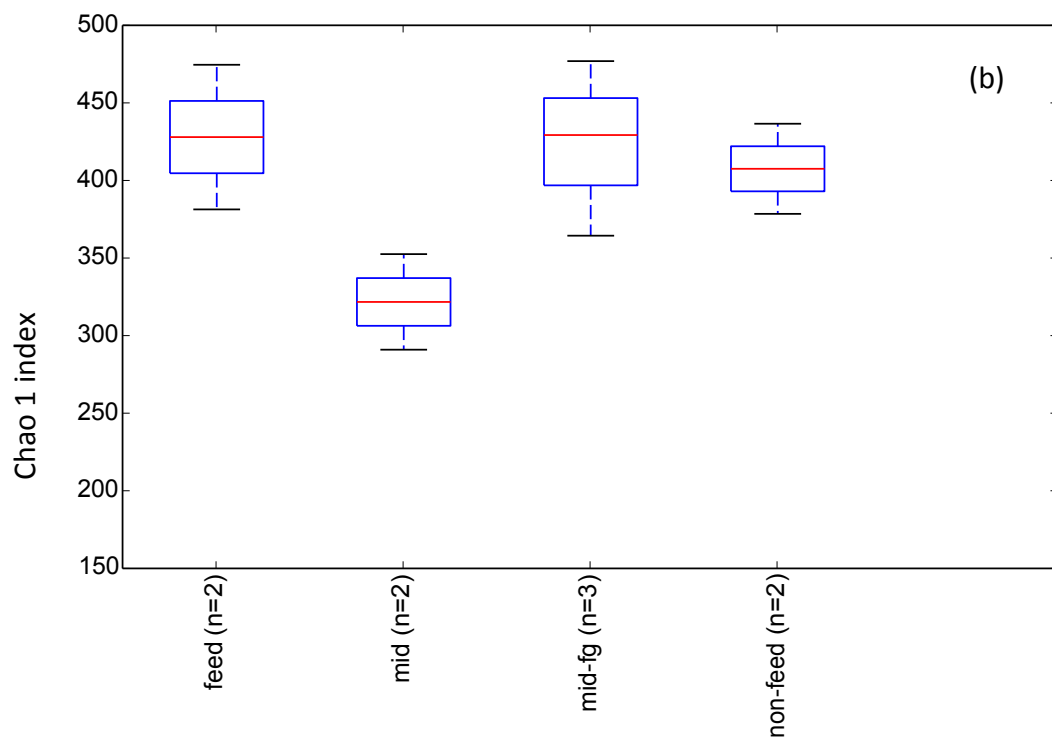

Supplement: Supplementary file 8 — Supplementary Figure 5 [file 41522_2017_21_MOESM8_ESM.pdf]

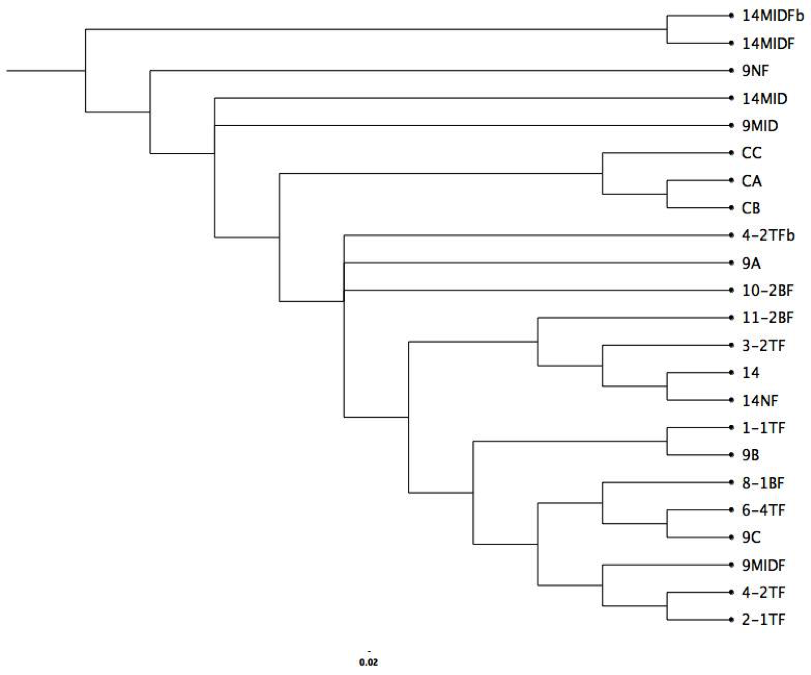

Supplement: Supplementary file 9 — Supplementary Figure 6 [file 41522_2017_21_MOESM9_ESM.tif]

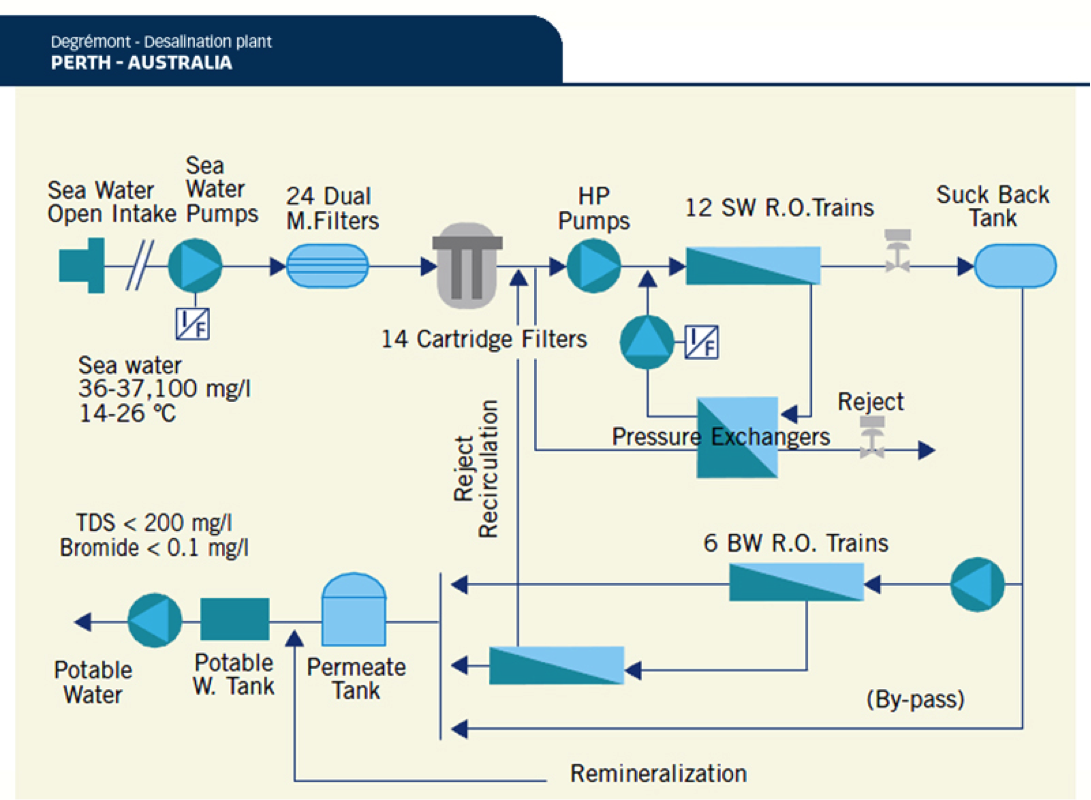

Supplement: Supplementary file 10 — Supplementary Figure 7 [file 41522_2017_21_MOESM10_ESM.tif]
